# Mapping multiple principles of parietal–frontal cortical organization using functional connectivity

**DOI:** 10.1007/s00429-018-1791-1

**Published:** 2018-11-23

**Authors:** Suhas Vijayakumar, Jerome Sallet, Lennart Verhagen, Davide Folloni, W. Pieter Medendorp, Rogier B. Mars

**Affiliations:** 1 Donders Institute for Brain, Cognition and Behaviour, Radboud University Nijmegen, 6525HR Nijmegen, The Netherlands; 2 Department of Experimental Psychology, Wellcome Centre for Integrative Neuroimaging, University of Oxford, 9 South Parks Road, Oxford, OX1 3UD United Kingdom; 3 Nuffield Department of Clinical Neurosciences, Wellcome Centre for Integrative Neuroimaging, Centre for Functional MRI of the Brain (FMRIB), John Radcliffe Hospital, University of Oxford, Headington, Oxford, OX3 9DU United Kingdom

**Keywords:** Cortical organization, Macaque, Parietal–frontal, Comparative, Connectivity fingerprints, Parcellation, Connectional families

## Abstract

Resting state functional connectivity has been promoted as a promising tool for creating cortical maps that show remarkable similarity to those established by invasive histological methods. While this tool has been largely used to identify and map cortical areas, its true potential in the context of studying connectional architecture and in conducting comparative neuroscience has remained unexplored. Here, we employ widely used resting state connectivity and data-driven clustering methods to extend this approach for the study of the organizational principles of the macaque parietal–frontal system. We show multiple, overlapping principles of organization, including a dissociation between dorsomedial and dorsolateral pathways and separate parietal–premotor and parietal–frontal pathways. These results demonstrate the suitability of this approach for understanding the complex organizational principles of the brain and for large-scale comparative neuroscience.

**Electronic supplementary material:**

The online version of this article (10.1007/s00429-018-1791-1) contains supplementary material, which is available to authorized users.

## Introduction

The ultimate goal of mapping the human brain is to understand the relationship between its organization and its function. However, to appreciate the evolution of this relationship, it is necessary to have more than a single species’ data point. Hence, one of the aims of comparative neuroscience is to map the brains of a range of species in terms of each of their underlying principles of organization (Striedter et al. 2014).

While this approach is almost prohibitively costly and laborious when using traditional histological methods, the advent of neuroimaging as a tool for mapping brain organization has opened up a range of possibilities. In the human brain, measures of both structure and function have been used to map areas based on their connectivity profiles (Gordon et al. 2016; Johansen-Berg et al. 2004; Kahnt et al. 2012; Sallet et al. 2013), myelin content (Glasser et al. 2014), and functional response profiles (Tsao et al. 2008). A recent effort combined these approaches to propose a novel map of the human brain (Glasser et al. 2016). Building on the success of such efforts, these techniques are also increasingly applied in non-human primates (Mars et al. 2014), allowing a direct comparison of cortical maps across species (Mantini et al. 2013; Mars et al. 2013; Neubert et al. 2014).

However, creating maps of cortical areas is only one step towards elucidating principles of cortical organization across species. A further requirement is to compare the architecture of inter-areal relationships. For instance, although the primate cortex consist of regions that have an individual profile of connections, the so-called connectivity fingerprint (Passingham et al. 2002), one can find connectional families of regions with similar connections at a higher level of description (Kötter et al. 2001). Moreover, some recent studies emphasize supra-regional aspects of connectional organization of the cortex over the connectivity of individual regions (Huntenburg et al. 2018; Jbabdi et al. 2013). Importantly, such topographical organizations have functional relevance for behavior (Marquand et al. 2018). Thus, to truly understand the relationship between cortical organization and behavior, it is imperative to be able to map inter-areal relationships as well as cortical territories.

The parietal–frontal system of the macaque monkey brain—one of the best understood systems in terms of primate cortical anatomy—provides an interesting case in point when examining overarching patterns of inter-areal organization. The most common description is that of multiple parallel parietal–frontal networks (Goldman-Rakic 1988), some of which might facilitate different aspects of the movement repertoire (Gharbawie et al. 2011). However, both the segregation of such information processing streams (Caminiti et al. 2015) and the convergence of parietal connections in parts of premotor cortex have been emphasized (Wise et al. 1997). At a higher level of description, a gradient can be seen in the inferior parietal cortex, where progressively more posterior regions connect to progressively more anterior frontal regions (Caspers et al. 2011), referred to as “core–shell organization”. Models concerned with describing whole-brain patterns of organization argue for an overarching dorsal–ventral distinction in the frontal–parietal organization (Hoshi and Tanji 2007; Pandya et al. 2015). In addition, a hierarchical organization of the frontal lobe (Badre and D’Esposito 2009), possibly with parallel medial and lateral hierarchies (Kouneiher et al. 2009), is evident in many functional models of cognition. Rather than competing, each of these models is concerned with a different level of description that has distinct explanatory value.

Here, we investigate whether we can use resting state fMRI to create a map of inter-areal organization of the macaque parietal–frontal system using resting state functional connectivity. We use a data-driven clustering technique to identify individual cortical areas in both parietal and frontal cortex following the approach of most mapping studies (Mars et al. 2011; Sallet et al. 2013), and subsequently investigate the inter-cluster organization across the two lobules using a hierarchical approach. This approach of divergent parcellation and convergent clustering allows to assess how well an observer-independent technique can describe principles of organization, which is crucial if the technique is later to be employed to study brains whose organization is less well-understood. We do note, however, that its simplicity and the use of group-level data, mean that our results should be taken as purely descriptive. To foreshadow the results, in agreement with previously established work, we find that parcellation-based resting state functional connectivity strongly resembles cytoarchitectonically established regions. Importantly, we show that this approach is able to find multiple, overlapping principles of organization, which suggests that it can be employed successfully as a technique for comparative neuroscience.

## Results

We parcellated both the frontal and the parietal cortex into regions that share a similar connectivity profile using a data-driven affinity propagation (AP) clustering algorithm (Frey and Dueck 2007). This algorithm both assigns each vertex to a cluster and identifies a single data point within each region—called exemplar—that best represents properties of the other data points of the region. To test for the principles of parietal–frontal organization, the correlation matrix of the frontal and parietal exemplars was clustered using hierarchical clustering, which iteratively groups together clusters into families of regions based on their connectivity with the other part of the cortex (frontal clusters based on parietal connectivity and vice versa). Finally, based on the results of this hierarchical clustering, we constructed an affinity matrix with eight frontal and seven parietal branches to explore parallel principles of cortical organization. We focus on validating the approach and discuss the results of the right hemisphere in the main text. Please see supplementary material for results of the left hemisphere.

### Parcellation of frontal and parietal cortex

**Fig. 1:**
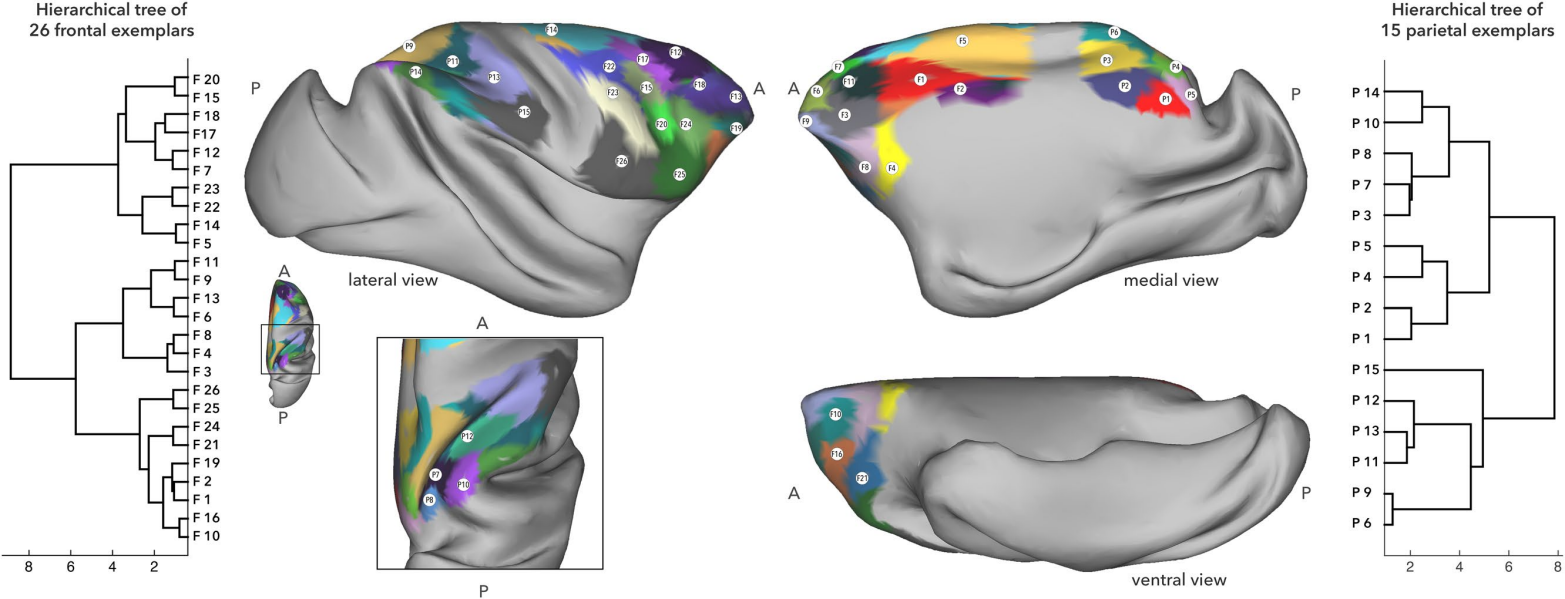
Results of AP clustering of the right parietal and the right frontal cortex based on their connectivity strength within the hemisphere, back projected onto an inflated macaque cortical surface. The cluster numbers mark the position of the exemplar as identified by the algorithm. Hierarchical clustering of the frontal exemplars based on their connectivity with the parietal exemplars is given on the left and that of the parietal exemplars based on their connectivity with the frontal exemplars is given on the right. *A* anterior, *P* posterior

Figure 1 illustrates the results of the parcellation, along with the hierarchical clustering of the exemplars. Parcellation of the frontal region of interest (ROI) based on its connectivity with the rest of the hemisphere resulted in 26 clusters. The clusters were identified in relation to cytoarchitectonically defined areas in the macaque frontal cortex based on the location of the exemplars and the boundaries of the parcels. In the rare case that the parcels were not spatially continuous, the location of the exemplar was used to determine its anatomical label. Parcellation of the parietal ROI resulted in 15 clusters that were assigned labels in the same way. While one could discuss the results by referring to each of the parcels simply with a number (Goulas et al. 2017), we have found that using labels based on their overlap with cytoarchitectonically defined areas (Neubert et al. 2014) makes our findings more tractable and helps communicate the functional relevance of the results. The labels are only meant to provide a heuristic. So, we note that the labels assigned to the following parcels need to be considered with caution. The goal of the study is not to identify the presence of a particular number of cytoarchitectonic areas, but to examine the principles of organization within the parietal–frontal system. The clustering is thus a pre-processing step to identify meaningful sub-regions within the data.

Table 1 presents a list of cluster numbers and their approximate location according to published cytoarchitectonic atlases. See supplementary material for their labeling based on the atlases of Lewis and Van Essen (2000), Paxinos et al. (2000), and Markov et al. (2011). On the medial wall, frontal clusters F1 and F2 overlapped with mid-cingulate cortex [cytoarchitectonic area 24 according to the atlas of Paxinos et al. (2000)]; clusters F3, F4, and F8 were in the territory of the peri-genual anterior cingulate cortex (ACC) (area 32, area 25). The dorsal posterior cluster F5 overlapped with supplementary motor area [SMA, termed F3 by Rizzolatti et al. (1998)]. On the lateral surface, clusters F14 and F22 matched the dorsal premotor cortex (PMd, area F2); clusters F23, F25, and F26, corresponded to the ventral premotor cortex (PMv, areas F4 and F5) and the macaque homolog of area 44 (Petrides et al. 2005). The peri-arcuate clusters F17, F15, and F20 overlapped with area 8. Clusters F7, F11, F12, and F18 overlapped with the dorsal prefrontal cortex (PFC) (area 9) and cluster F13 with dorsolateral PFC (area 46). Cluster F24 corresponded to the ventral part of lateral PFC. While clusters F6 and F9 were in the territory of frontopolar cortex (area 10), clusters F10, F16, F19, and F21 corresponded to orbitofrontal cortex (area 11, area 13, and area 47/12).

**Table 1:** Description of parcels in terms of cytoarchitectonic anatomical maps

Exemplar number	Anatomical region (most overlapping areas)
Frontal cortex	
F1, F2	Mid-cingulate cortex (area 24)
F3, F4, F8	Peri-genual ACC (areas 32, 25)
F14, F22	Dorsal premotor (F2)
F5	Supplementary motor area (F3)
F23, F26, F25	Ventral premotor (F4, F5, area 44)
F24	Ventral PFC (area 12)
F18, F12, F7, F11	Dorsal PFC (area 9)
F13	Dorsolateral PFC (area 46)
F17, F15, F20	Peri-arcuate cortex (area 8)
F6, F9	Frontopolar cortex (area 10)
F10, F16, F19, F21	Orbitofrontal cortex (areas 11, 13)
Parietal cortex	
P1, P2, P3, P6	Medial SPL (PGm, area 31, PEci, PE)
P4, P5	Anterior bank of POS (PEc, PO)
P9, P11, P13	SPL (anterior PE, posterior area 2, area 5 and 7)
P15, P14, P10	IPL (PF, PG, Opt)
P7, P8	Posterior IPS (VIP, PEa)
P12	Anterior IPS (VIP, AIP)

*ACC* anterior cingulate cortex, *PFC* prefrontal cortex, *SPL* superior parietal lobule, *POS* parieto-occipital sulcus, *IPL* inferior parietal lobule, *IPS* intra-parietal sulcus

On the medial wall of the parietal cortex, two ventral clusters P1 and P2 overlapped with area PGm and area 31. Posterior to these were clusters P4 and P5, on the anterior bank of the parieto-occipital sulcus (POS), of which the more dorsal cluster, cluster P4, overlapped with PEc (Saleem and Logothetis 2012). Of two more dorsal clusters on the medial wall, cluster P3 was located mostly within the posterior end of the cingulate sulcus (overlapping with area PEci) and cluster P6 was located superior to that (overlapping with area PE). Clusters P9, P11 and P13 occupied the anterior part of the intra-parietal sulcus (IPS) and the superior parietal lobule (SPL), of which the anterior clusters P13 and P11 largely overlapped with the most anterior parts of PE and the posterior part of area 2, while the posterior cluster P9 followed area 5 and 7 along the dorsal/medial part of the IPS. On the inferior parietal lobule (IPL), an anterior cluster P15 overlapped with area PF; more posterior clusters P14 and P10 overlapped with parietal area 7a (Cavada and Goldman-Rakic 1989) with cluster P14 confined to PG, and cluster P10 overlapping with Opt as described by Paxinos et al. (2000). Two additional clusters P7 and P8 were buried in the posterior part of the IPS, overlapping with VIP and PEa (Saleem and Logothetis 2012). Cluster P12, in the anterior part of IPS, spanned the territory of VIP and AIP.

### Principles of hierarchical organization

**Fig. 2:**
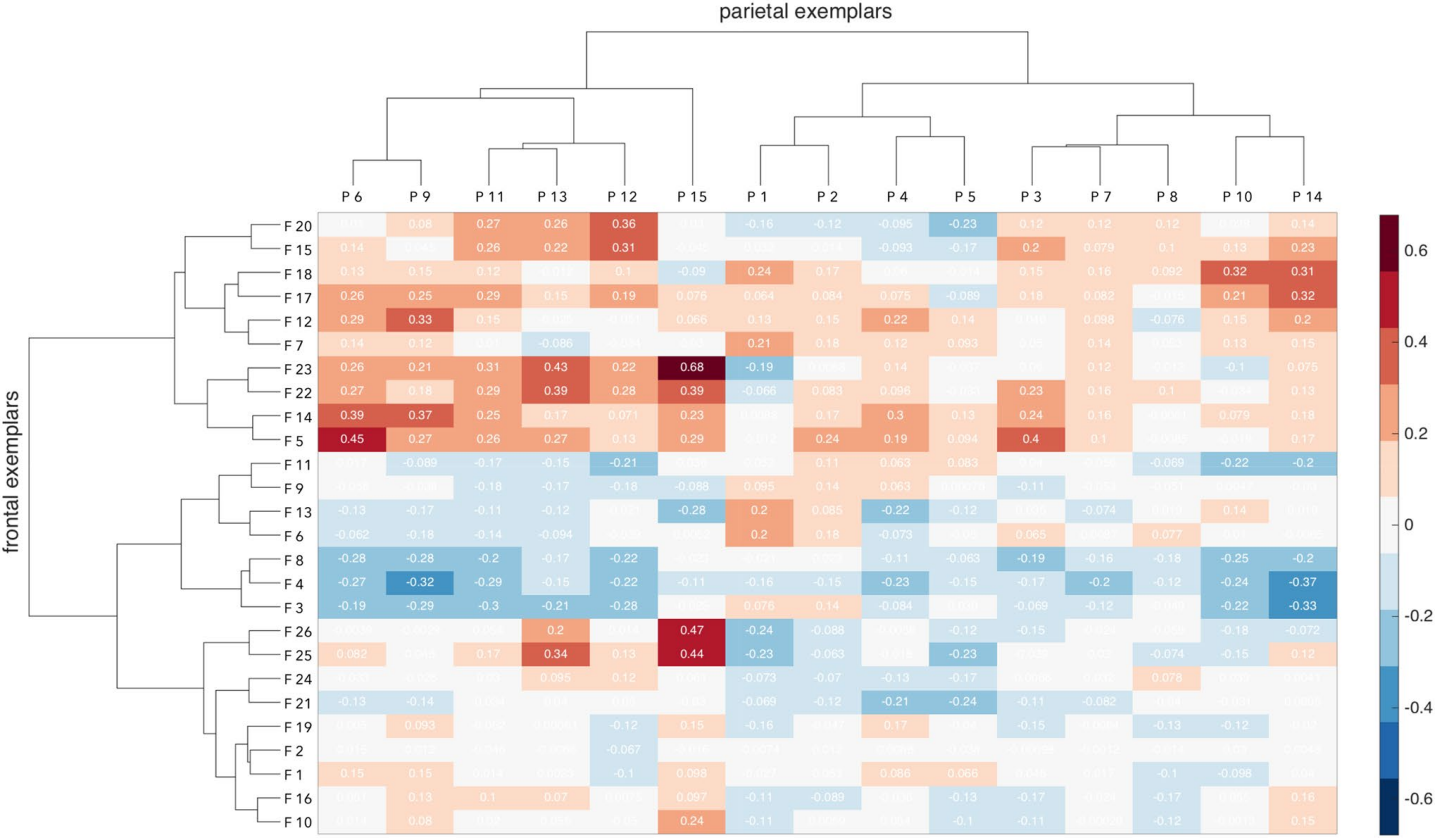
Connectivity fingerprints of the frontal exemplars (rows) and the parietal exemplars (columns), along with their hierarchical clustering outline. Values indicated in each cell correspond to the correlation value of the corresponding frontal and parietal exemplars, as shown in Fig. 1. Color bar ranges from the lowest to the highest correlation value of the exemplars

The connectivity between all frontal exemplars and all parietal exemplars is displayed in Fig. 2. All subsequent hierarchical analyses are perfromed using this connectivity matrix as their basis.

#### Frontal cortex

**Fig. 3:**
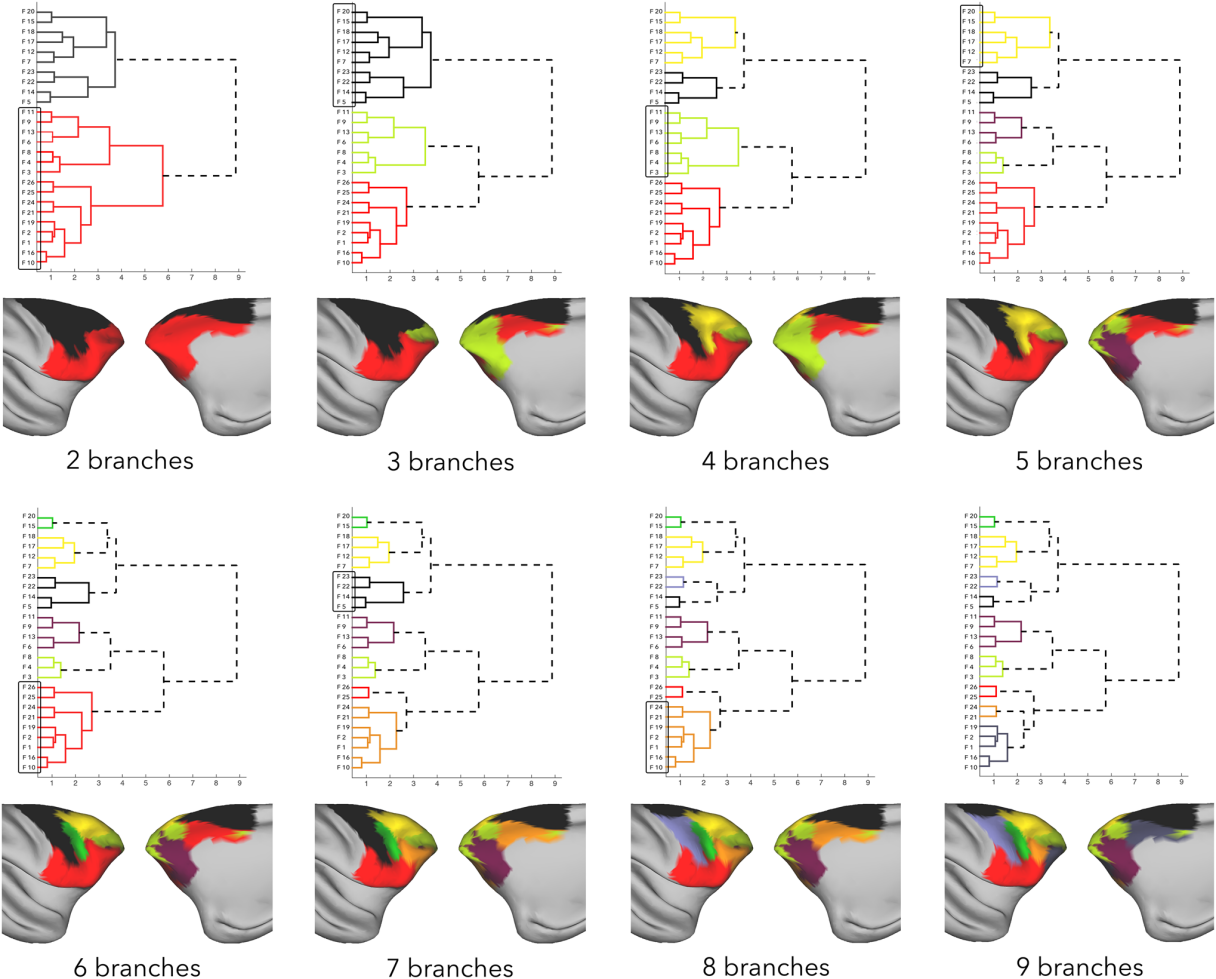
Hierarchical clustering results of the frontal exemplars based on their parietal connectivity strength. Exemplars (by extension, parcels) that belong to a branch are overlaid on the surface of the cortex in the same color, and the branch that splits in the following step is marked with a box

Figure 3 shows results of hierarchical clustering of the frontal cortex based on its connectivity with the parietal cortex. The initial branching into two branches dissociated regions with strong and widespread connectivity with parietal cortex and regions with weak or very focal parietal connectivity. The regions with strong connectivity were further subdivided (see change from 3 to 4 branches in Fig. 3) into dorsal premotor regions with strong connectivity to anterior IPL and dorsal SPL, and the dorsal PFC and peri-arcuate regions with strong connectivity to regions in posterior IPL and posterior IPS. These latter regions were subdivided into dorsal PFC and peri-arcuate, based on their preferential connectivity to posterior IPL and posterior IPS, respectively (5–6 branches). Due to their overall similarity in connections with the parietal cortex, the dorsal premotor regions only split into the medial–dorsal premotor part (including SMA) and a lateral part in the later stages of hierarchical clustering (7–8 branches). They were distinguished by SMA and the most dorsal part of PMd showing connectivity with dorsal superior parietal lobe (SPL), and the lateral premotor area having comparatively stronger connections with the anterior segments of the parietal cortex.

Regions in the frontal cortex with low or negative connectivity with the parietal cortex were divided into the peri-genual anterior cingulate (ACC) and the frontopolar regions on the one hand, and ventral premotor and prefrontal regions, and cingulate motor areas (CMA) on the other (2–3 branches). This dissociation was driven by the first branch’s regions showing very weak or no connectivity with parietal cortex, whereas ventral frontal and CMA showed focal connectivity with the anterior most part of inferior parietal lobule (IPL). These latter regions were in turn subdivided based on their specific connectivity with IPL—ventral premotor (PMv) and area 44 with strong anterior IPL connectivity and ventral PFC and CMA with weaker connectivity to IPL (6–7 branches). Ventral PFC and CMA were finally subdivided based on their connectivity with posterior IPL (8–9 branches). Posterior part of the ventral PFC showed selective connectivity with posterior IPL, while the anterior part of the ventral PFC and CMA showed no particular peak in connectivity with the parietal cortex. Peri-genual ACC and frontopolar regions were subdivided into ventromedial regions with weak connectivity with the parietal cortex in general and frontal polar regions (including area 9/32 on the medial side) (4–5 branches).

Following the progression through the hierarchy, we observed that from the 9 branch solution onwards, branches start to disintegrate into individual regions, sometimes even resulting in spatially discontinuous branches (clusters F21 and F24, in this case). As our aim was to investigate the organization in terms of connectional families, and not connections of individual regions, the frontal cortex was clustered into 8 branches to capture the required level of detail of functional families. We demonstrate the approach using affinity matrices constructed with (frontal, parietal) branches of (2,2), (4,4), (7,6), and (8,8) in the supplementary material (section titled “affinity matrices”).

#### Parietal cortex

**Fig. 4:**
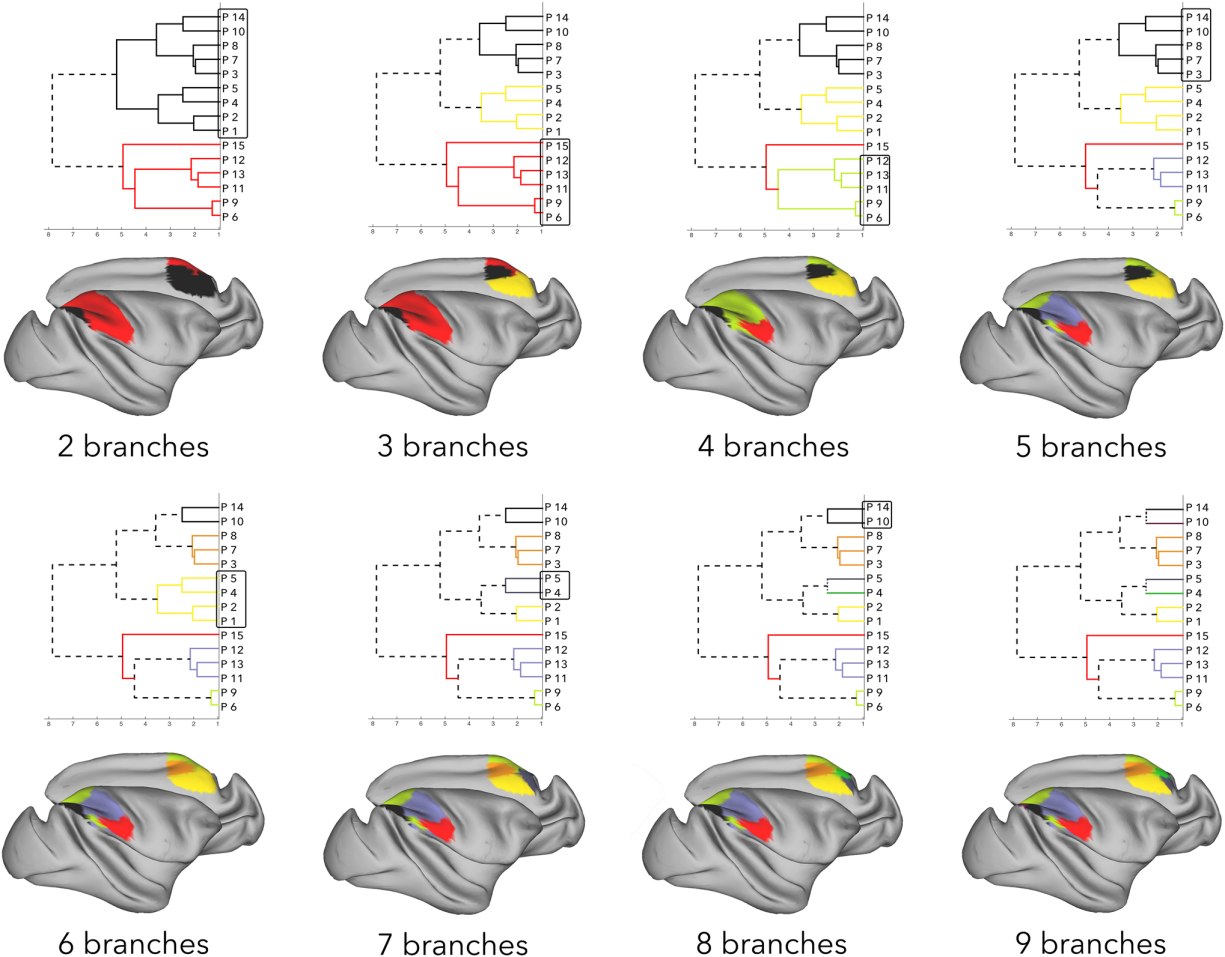
Hierarchical clustering results of the parietal exemplars based on their frontal connectivity strength. Exemplars (by extension, parcels) that belong to a branch are overlaid on the surface of the cortex in the same color, and the branch that splits in the following step is marked with a box

Figure 4 shows the results of hierarchical clustering of the parietal cortex based on its connectivity with the frontal cortex. The main division of parietal cortex into two branches was driven by regions having strong connectivity to the frontal cortex [Fig. 2, first 10 branches (clusters F20–F5)], and regions having comparatively weaker connectivity with the frontal cortex [Fig. 2, last 16 branches (clusters F11–F10)]. The regions with stronger connectivity contained anterior IPL and SPL, which was driven by anterior IPL having strong connectivity with ventral premotor regions, while SPL has stronger connectivity with PMd, SMA, and the peri-arcuate regions (3–4 branches). SPL was further divided into regions on the anterior SPL and IPS, that preferentially showed connectivity with the peri-arcuate regions, and posterior SPL regions that showed comparatively stronger connectivity with PMd, SMA, and dorsal PFC (4–5 branches).

The regions with comparatively weaker connectivity to the frontal cortex mostly showed similar connectivity with the dorsal PFC. However, posterior parts of IPL and IPS, and PEci connected preferentially to dorsal–caudal regions of frontal cortex including PMd, while area 31, PGm and PO had preferential connectivity with the more rostral parts of the frontal cortex, the frontal polar region (2–3 branches). The regions with PMd connectivity were further divided based on a rostro-caudal connectivity dissociation (branches 5–6). Regions PEci, VIP, and PEa showed weak PMd connectivity. VIP and PEa also showed weak SMA connectivity, whereas PEci revealed strong connectivity with SMA. The posterior IPL, PG, and Opt regions showed comparatively stronger connectivity with the dorsal PF cortex. As these latter three regions share similar connectivity fingerprints, they were not separated/distinguished until hierarchical clustering reaches a 9-branch dissociation, where posterior IPL and PG were separated from Opt due to their connectivity with the peri-arcuate.

The regions with rostral connections—area 31, PGm and PO—were further divided based on how strongly they connected with the frontal polar region (branches 6–7). Area 31 and PGm, with stronger connectivity to both dorsal PF and frontal polar cortex were grouped under the same branch, whereas PO was separated for having mostly dorsal PF cortex connectivity.

Just as in the frontal hierarchical clustering solutions, branches start resolving in to individual regions from 8 branch solution onwards. Hence, to retain and examine the connectivity profiles of families of regions, the 7 branch solution was used to subsequently construct the affinity matrix.

### Principles of large-scale organization

Hierarchical clustering results provided insight into the organization of the cortex at multiple levels. At a specific level of branching, in which regions with similar connectivity profiles clustered together, we were able to identify multiple organizational principles driving the large-scale organization of the frontal–parietal network.

#### Dorsal–ventral organization

**Fig. 5:**
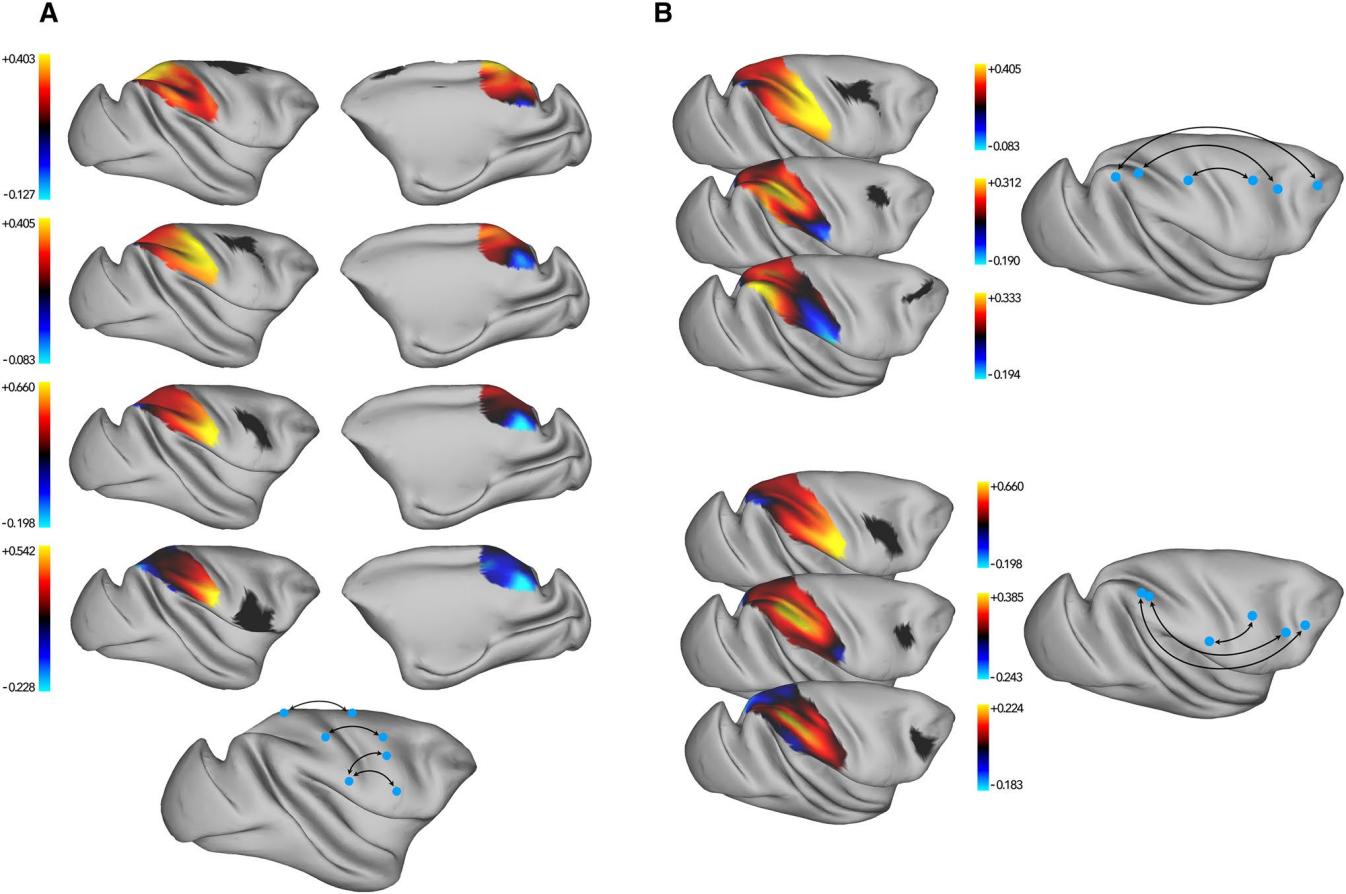
Regions in the frontal cortex and their corresponding exemplar’s connectivity with the parietal cortex. Black regions indicate seed areas in the frontal cortex, with the blue–red–yellow colors indicating increasing connectivity with each parietal vertex. Maximum connectivity vertices and schematic representation of the organization of these connections are also shown. **a** Dorsal–ventral organization of parietal connectivity with the premotor cortex. **b** Rostral–caudal or core–shell organization of parietal connectivity with the lateral frontal cortex, illustrated separately for dorsal (top) and ventral (bottom) frontal regions. Color bars represent the parietal connectivity correlation values of the exemplars of these seed regions. Note that the color of the ROI does not represent its within-region connectivity, but just a representation of the region

One of the main principles of organization described for the parietal–premotor pathways is a distinction between the dorsal or medial pathway connecting the parietal reach region at the back of the SPL with the dorsal premotor cortex, and a ventral or lateral pathway connecting the inferior parietal lobule with the ventral premotor cortex (Hoshi and Tanji 2007; Rizzolatti and Matelli 2003; Tanné-Gariépy et al. 2002). To explicitly test whether a dorsal–ventral gradient in parietal–premotor connectivity was evident in our data, we plotted the connectivity of the four most posterior frontal clusters with the whole of parietal cortex (Fig. 5a). We found that connectivity strengths of the four clusters mirrored their dorsal–ventral dissociation in the parietal cortex. PMd connected strongly with dorsal SPL; middle premotor regions connected with the posterior IPL and IPS; PMv connected strongly with the anterior IPL. This confirms the dorsal–ventral organization of the premotor cortex, based on receiving information from parallel visuomotor streams.

#### Core–shell organization

A second major organization principle of the frontal–parietal networks is that the regions on either side of the central sulcus have highest connectivity with one another, and so do the parietal and frontal regions progressively farther away from the sulcus. This is known as a ‘core–shell’ organization. Using a similar strategy as above for the dorsal–ventral organization, we selected clusters along a caudal–rostral gradient in the frontal cortex and plotted the connectivity with the whole of parietal cortex (Fig. 5b). We selected two series of regions, one dorsal to the principal sulcus and one ventral to it. As shown, as the ROIs progress along the posterior to anterior axis, the peak connectivity in the parietal cortex changes from anterior to posterior, obeying the “core–shell” organization.

#### Parallel frontal–parietal connections

Anatomical studies have established that the premotor cortex receives information from the parietal cortex through separate visuomotor pathways. Averbeck et al. showed that this organization can be described in terms of families of frontal regions preferentially connecting to families of parietal regions (Averbeck et al. 2009; Caminiti et al. 2017). To infer the existence of such an organization using functional connectivity data, we investigated the parietal–frontal functional connectivity at a single level of description. Based on the results discussed above, we chose a level of seven parietal branches and eight frontal branches. This level of description captures the grouping of areas into functionally distinct parcels, such as the dorsal/medial premotor cortex, ventral premotor and caudal ventral frontal cortex, ventral arcuate, dorsal PFC, and peri-genual ACC.

**Fig. 6:**
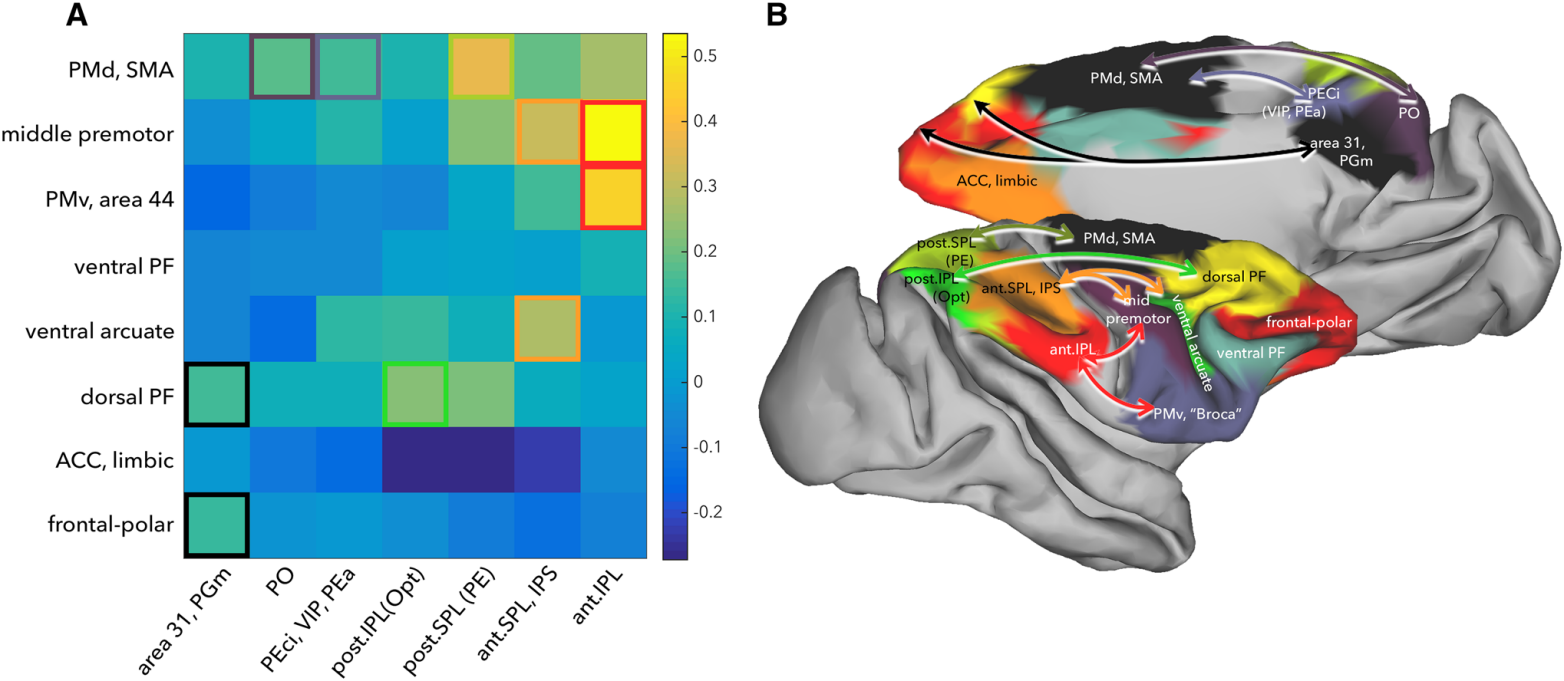
**a** Affinity matrix of the 8 frontal hierarchical branches (rows) with that of the 7 parietal branches (columns), indicating the average connectivity strength of the exemplars within each branch. **b** The branches are overlaid on a macaque brain along with arrows representing the peak connectivity between branches

We created an affinity matrix that describes the average connectivity strength of the exemplars of families of frontal regions connecting to the families of the parietal region (Fig. 6a). The maximum connectivity strengths in this affinity matrix indicate some of the main parietal–frontal pathways (Fig. 6b). It was successful in capturing the dorsal “reach” system involving the PMd, SMA and the posterior–medial SPL (Caminiti et al. 1996; Marconi 2001), the lateral “grasp” system involving the PMv and anterior IPL (Bonini et al. 2012; Gharbawie et al. 2011), and the oculomotor intention and attention system consisting of the dorsal PFC (area 8, 46) and the posterior IPL (Andersen and Cui 2009). These results support the presence of parallel information processing streams, characterized by stronger connectivity between regions equidistant from the central sulcus (Caminiti et al. 2017; Genon et al. 2017).

## Discussion

We sought to map the organizational principles linking the parietal and frontal cortex in the macaque monkey using resting state fMRI. While previous studies using functional connectivity measures and clustering algorithms have mainly focused on mapping cortical regions, we aimed to extend this approach to identify the principles guiding cortical organization at multiple levels of description—from the organization of individual functional areas to the nature of large-scale circuits. Accordingly, we used a data-driven clustering approach to parcellate the parietal and frontal lobules based on whole-brain connectivity, and then used hierarchical clustering to find families of regions between the two lobules based on parietal–frontal connectivity. The clusters resulting from the initial parcellation unsurprisingly showed remarkable similarity with areas previously identified by cytoarchitecture (Paxinos et al. 2000). The hierarchical clustering allowed us to investigate the parietal–frontal system based on their inter-areal connectivity, which was able to establish that the higher-level groupings of frontal clusters show preferential connectivity to particular parietal groupings. On a larger scale, the affinity matrix between groupings revealed two concurrent principles of organization: a dorsal–ventral organization and a core–shell organization along the anterior–posterior axis.

One of the strongest dissociations in the data was the initial subdivision of the frontal cortex into a superior branch of regions showing widespread parietal connectivity, and a ventral/medial branch showing either low or very restricted connectivity with parietal cortex. Only then did these branches dissociate into caudal premotor clusters and rostral prefrontal clusters. The dorsal/ventral dissociation of the premotor cortex and their respective connectivity with superior and inferior parietal cortex mimicked the dissociation between the dorsomedial stream consisting of superior parietal areas PO and MIP and the dorsal premotor cortex, and the dorsolateral stream consisting of the AIP and anterior IPL and ventral premotor cortex (Rizzolatti and Matelli 2003).

Areas of the dorsal prefrontal cortex and areas around the arcuate sulcus also showed strong connectivity with the parietal cortex, in particular with the IPS and the posterior IPL. The ventral and medial part of the prefrontal cortex showed much weaker connectivity with the parietal cortex, with parietal connectivity of orbitofrontal cortex mostly focused on the anterior IPL and parietal connectivity of medial prefrontal clusters focused on medial parietal cluster. The lack of widespread parietal connectivity of these clusters is consistent with observations that these regions receive relatively more white matter fibers from the temporal cortex and the limbic system (Carmichael and Price 1995; Folloni et al. 2017).

We concentrated our discussion on the right hemisphere of the macaque, but analogous analyses were performed in the left hemisphere which are reported in the supplementary material. Importantly, we were able to identify the dorsal–ventral organization of the premotor cortex, as well as the core–shell principle of organization in the parietal–frontal networks in both the hemispheres. However, at underlying levels of description there were noticeable differences, which meant the affinity matrices showed differences in the strength of the organizational principles between the hemispheres. Differences in the branches of the hierarchical clustering analysis and consequently in the affinity matrices were perhaps driven by the differences in the parcellation results. For instance, the right hemisphere contained three exemplars from the mid-cingulate region on the medial, while the left hemisphere contained one. The premotor cortex contained four exemplars in the right, and three in the left. Since parcellation results were based on within-hemisphere functional connectivity, we acknowledge that there are variations in the strength of the organizing principles between hemispheres that deserve future study.

Resting state fMRI in combination with clustering algorithms has previously been used successfully to parcellate areas of the cerebral cortex (Goulas et al. 2017; Kahnt et al. 2012), as well as to understand weighted contributions of such regions to cortical networks (Beckmann et al. 2005; Smith et al. 2013). In effect, these approaches are aimed at studying cortical organizations at different levels, but only their extreme ends. As alluded to in the introduction, connectivity can be described in terms of the connectivity fingerprint of individual regions, connectional families of regions, and larger-scale networks. The present results demonstrate that, by investigating different levels of hierarchical clustering branches of the parietal and frontal cortex, one can identify organization at all these levels. We have previously shown that resting state functional connectivity is able to identify the main frontal–parietal connections in both humans and macaques (Mars et al. 2011), but the current work further demonstrates that the same data reflect multi-scale organization of these cortices.

In their seminal paper on the importance of brain connectivity in understanding cortical organization, Passingham et al. (2002) explicitly linked a cortical area’s connectivity fingerprint to its functional role. They showed that regions that group together based on their connectivity also tend to group together based on their involvement in specific functions. Moreover, this relationship might scale, with connectional families of areas and families of areas based on similarity in function showing similar groupings. Caminiti et al. (2017) illustrated this point when they delineated the macaque parietal–frontal pathways based on a meta-analysis of tracer data in the macaque and then explicitly linked the pathways to different motor and attentional functions. For instance, they were able to identify dorsal reach system involving the SPL and medial and dorsal premotor areas, and the lateral grasp system consisting of the anterior IPL and ventral premotor and frontal regions, but also the oculomotor and attentional system involving the posterior IPS and dorsal prefrontal cortex. These functional systems were also confirmed in our current data and can be identified based on the affinity matrix as described above (Fig. 6). Importantly, the hierarchical analysis of the resting state data mimics the functional relationship of regions. For instance, the dorsal SPL-premotor network separates quite early on from the dorsal prefrontal and peri-arcuate systems that both have been attributed roles in attentional behavior. These networks then separate out themselves at a later stage, and indeed their roles in attention might be dissociated, with peri-arcuate regions more involved in top-down orienting of attention and the dorsal PFC in providing the context of an action based on abstract goals (Passingham and Wise 2012).

A more recent view of the functional organization of the parietal–premotor system emphasizes parallel networks involved in the control of different ethological movement categories, such as a defensive posture, eating behavior, manipulation in near space, and locomotion (Graziano 2016; Kaas et al. 2011). This type of organization is particularly interesting from a comparative perspective, as it would make predictions of how parietal–premotor systems might be differentially developed to suit the niche of a particular animal. It has been argued that human motor behavior, for instance, might rely on a novel pathway (Peeters et al. 2009), but an alternative view would be that it relies on an elaboration of the existing primate pathway for nearby object manipulation (Hecht et al. 2013). The present approach is able to dissociate these pathways in a data-driven manner, allowing future work to test the hypothesis that there is an additional pathway in the human brain. Although diffusion MRI tractography studies have shown that the major white matter pathways connecting the frontal and parietal cortex can also be identified in the human brain (Makris et al. 2005; Thiebaut de Schotten et al. 2012), it has also demonstrated some lateralizations that are suggested to be specific to the human (Thiebaut de Schotten et al. 2011; Howells et al. 2018) as well as some more fundamental differences in the projections of certain tracts (Eichert et al. 2018; Rilling et al. 2008). However, to our knowledge, a full analysis of human parietal–frontal organization along the lines of the current study, which sheds light on unique aspects of our parietal–frontal organization has not been performed yet and is the topic of ongoing research in our lab.

While the various models discussed above predict various aspects of our data, it is tempting to consider whether there is an overarching theory of cortical organization that can explain the various levels of description within a single framework. One model that has this potential is the so-called “dual origin model”, originally described by Sanides (1964) and recently elaborated upon by Pandya et al. (2015). According to this model, trends of neocortex expansion and differentiation emerge from two allocortical sources. The hippocampal differentiation includes the medial wall of the hemisphere, and superior parts of the parietal, dorsal premotor, and dorsal frontal cortex; the pyriform differentiation includes ventral premotor and prefrontal including orbital cortex and temporal pole. The dorsal/ventral dissociation we observed may then be the result of two separate expansions, leading to two mostly dissociated processing streams. According to the dual origin model, this dorsal–ventral dissociation is complemented by a subdivision of each part of sensory and motor cortex into parallel lines of cortical areas, ranging from least differentiated belt regions to core regions and then to maximally differentiated or primary root areas. Long-range connections between cortical systems are thought to occur between areas that are at similar stages of cortical differentiation, which might start to explain the core/shell organization of parietal–frontal connectivity we observed. Although the dual origin model was originally proposed based on observations in reptiles, monotremes, and marsupials, recent treatment is fully based on observations in the macaque monkey (Pandya et al. 2015). The results of the present study are thus largely consistent with these suggestions, although it should be noted that the cingulate areas are originally grouped with ventral premotor regions in our data, rather than with the dorsal premotor regions as predicted by the theory (Pandya et al. 2015). This could, however, be due to the generally weak parietal connectivity of the cingulate regions.

A related theory, proposed recently by Buckner and Krienen (2013) and referred to as the “tethering hypothesis”, also evokes primary cortices in explaining the architecture of long-range connections. According to this theory, signaling gradients and extrinsic activity from primary areas place strong constraints on the developing cortex. When the cortical sheet expanded dramatically, most of the emerging novel territory was distant from these constraints and emerged as association cortex that tended to wire together forming multiple parallel circuits as opposed to the hierarchical circuits seen among primary areas. Again, this hypothesis predicts some of features of parietal–frontal connectivity we observed.

These hypotheses, although intriguing, await further verification. While traditional techniques such as invasive tract tracing and cytoarchitecture have contributed immensely to our knowledge of brain anatomy and organization, they are labor intensive, time consuming, and cannot be used to study the brain of living animals, including humans. Moreover, elucidating principles of organization often requires integration of information from multiple studies performed on different individual animals (Averbeck et al. 2009), rather than studying whole-brain organization within the same group of subjects. Here, we demonstrate that resting state functional connectivity and clustering techniques can go beyond mapping cortical areas and can successfully identify the principles of large-scale brain organization. As functional MRI data are now obtained in an increasing number of species, including macaque and other Old World (Salinas and Szabó 2015) and New World (Ghahremani et al. 2016) monkeys and other mammals such as the dog (Kyathanahally et al. 2015), the ferret (Zhou et al. 2016), and rodents (Grandjean et al. 2017; Ortiz et al. 2018), we argue that this approach has the potential for large-scale comparative mapping of principles of brain organization across the mammalian order.

## Materials and methods

### Data acquisition

Macaque fMRI and anatomical scans were collected from seven healthy macaque monkeys (*Macaca mulatta*, seven males, median age = 4.39 years; median weight 8.3 kg). Protocols for animal care, magnetic resonance imaging, and anaesthesia were carried out under the authority of personal and project licenses in accordance with the UK Animals (Scientific Procedures) Act 1986 (ASPA).

Anaesthesia was induced using intramuscular injection of ketamine (10 mg/kg) either combined with xylazine (0.125–0.25 mg/kg) or with midazolam (0.1 mg/kg) and buprenorphine (0.01 mg/kg). Macaques also received injections of atropine (0.05 mg/kg intramuscularly), meloxicam (0.2 mg/kg intravenously) and ranitidine (0.05 mg/kg intravenously). Anaesthesia was maintained with isoflurane. The anesthetized animals were either placed in an MRI compatible stereotactic frame (Crist Instrument Co., Hagerstown, MA, USA) or resting on a custom-made mouth mold (Rogue Research, Mtl, QC, CA, USA). All animals were then brought into a horizontal 3T MRI scanner with a full-size bore. Resting state fMRI data collection commenced approximately 4 h after anaesthesia induction, when the peak effect of ketamine was unlikely to be still present. In accordance with veterinary instruction, anaesthesia was maintained using the lowest possible concentration of isoflurane gas. The depth of anaesthesia was assessed using physiological parameters (continuous monitoring of heart rate and blood pressure as well as clinical checks for muscle relaxation prior to scanning). During the acquisition of the MRI data, the mean inspired isoflurane concentration was 1.125% (ranging between 1.025% and 1.458%) and the mean expired isoflurane concentration was 1.083% (ranging between 1 and 1.317%). Isoflurane was selected for the scans as resting state networks have previously been demonstrated to closely match known anatomical circuits using this agent (Neubert et al. 2014; Vincent et al. 2007). Slight individual differences in physiology cause slight differences in the amount of anaesthetic gas concentrations needed to impose a similar level of anaesthesia on different monkeys.

All but one animal were maintained with intermittent positive pressure ventilation to ensure a constant respiration rate during the functional scan; one macaque was breathing without assistance. Respiration rate, inspired and expired CO_2_, and inspired and expired isoflurane concentration were monitored and recorded using VitalMonitor software (Vetronic Services Ltd., Devon). In addition to these parameters, core temperature was monitored using a Opsens temperature sensor (Opsens, Quebec, Canada) and pulse rate, and SpO_2_ (> 95%) was monitored using a Nonin sensor (Nonin Mediacal Inc., Minnesota, USA) throughout the scan.

A four-channel phased-array radio-frequency coil in conjunction with a local transmission coil was used for data acquisition (Dr. H. Kolster, Windmiller Kolster Scientific, Fresno, CA, USA). Whole-brain blood oxygen level-dependent (BOLD) fMRI data were collected for 1600 volumes from each animal (except for one with 950 volumes), using the following parameters: 36 axial slices, in-plane resolution 1.5 × 1.5 mm, slice thickness 1.5 mm, no slice gap, TR = 2280 ms, TE = 30 ms. Structural scans with a 0.5 mm isotropic resolution were acquired for each macaque in the same session, using a T1-weighted MP-RAGE sequence.

### Pre-processing

To date, most neuroimaging studies have focused either on generating detailed cortical maps, or on understanding topological principles, but not both. The approach has also been to validate the results across individuals (Margulies and Petrides 2013). But non-human primate fMRI data is often of variable quality when compared to human studies (Milham et al. 2017). Therefore, we used iterative PCA to analyze the variance in the data that is common to various subjects and not due to individual variation (Smith et al. 2014). The method has also been recently applied to tractography data (O’Muircheartaigh and Jbabdi 2018). Here, we use a standard brain mapping approach of clustering cortical territory into cortical regions based on functional connectivity data and then investigate inter-areal principles of organization from those results. We aim to test whether this simple approach allows us to infer known principles of anatomical organization and thus establish it as a tool suitable for comparative neuroscience.

The following pre-processing steps were performed using tools from FSL (https://fsl.fmrib.ox.ac.uk/), the Human Connectome Project Workbench (http://www.humanconnectome.org/software/connectome-workbench), and the in-house MR Comparative Anatomy Toolbox (Mr Cat, http://www.neuroecologylab.org).

Individual T1-weighted structural scans were corrected for the RF field bias and segmented into cerebrospinal fluid (CSF), gray matter, and white matter using a modified implementation of FMRIB’s Automated Segmentation Tool (FAST), brain extracted using a modified implementation of FSL’s Brain Extraction Tool (BET), registered to the F99 template (Van Essen 2002) using affine linear and subsequent nonlinear registration.

BOLD functional data were skull-stripped and co-registered to the structural data using linear registration. Data were filtered to remove scanner drifts and the dominant time course of CSF and white matter were regressed out before warping the functional data to F99 standard space. The functional data were then projected to the cortical surface using the Connectome Workbench. The resulting surface-projected time series data were normalized and smoothed along the cortical surface (*σ* = 3 mm).

Surface-time series data of all individuals were grouped using the MELODIC’s Incremental Group-PCA (MIGP) (Smith et al. 2014) resulting in one group-level time series dataset, which was then converted to a dense connectome file that stored the correlation value of each vertex with every other vertex in the ipsilateral cortex. We do note, however, that its simplicity and the use of group-level data, mean that our results should be described as descriptive.

### Parcellation of the frontal and parietal cortex

As the final step of pre-processing, we identified parcels present in frontal and parietal cortex. To this end, we submitted regions of interest (ROIs) covering the frontal and the parietal cortex to connectivity-based parcellation (cf. Johansen-Berg et al. 2004; Mars et al. 2011). A comprehensive description of our approach is provided below. In essence, we grouped together those vertices that have a similar connectivity profile with all the vertices in the ipsilateral hemisphere into a distinct parcel. This yields clusters consistent with the resolution of the data, ensuring we do not ignore distinct connectivity patterns present in the data or try to identify clusters that are too small to meaningfully relate to existing cytoarchitectonic parcellations of the macaque cortex. Although the goal here was not to define all of the cytoarchitechtonic regions that have been established in the macaque cortex, previous work has shown that the parcels derived from resting state fMRI data respect existing anatomical boundaries (Goulas et al. 2017; Neubert et al. 2014). Consistency of the results can be judged by similar analyses performed using a slightly different parietal ROI, as discussed in the supplementary material (section “alternate parietal ROI”).

Frontal and parietal ROIs were drawn based on sulcal boundaries of the cortex using Workbench. As shown in supplementary Fig. 5, the frontal ROI encompassed the lateral and medial aspects of the frontal lobe, including the premotor and the prefrontal cortex. The posterior border traced the anterior bank of the central sulcus one-third of the way along the precentral gyrus, continuing on the dorsal bank of the lateral sulcus at the ventral end laterally and the extension of the central sulcus/precentral gyrus border on the medial side. The ventral border of the ROI on the medial surface was formed by the ventral edge of the cingulate cortex. The anterior ventral border was formed by drawing a straight line between the posterior end of the subcallosal cingulate cortex on the medial surface that extended across the caudal orbital surface of the cortex, joining a projected extension of the lower limb of the arcuate sulcus. The ventral border on the lateral side traced the dorsal bank of the lateral sulcus, until it intersected a projected extension of the central sulcus. The posterior ventral border consisted of the ventral border of the ventral premotor cortex.

The anterior border of the parietal ROI traces the posterior bank of the central sulcus two-thirds of the way along the postcentral gyrus. The posterior border of the lateral side of the parietal ROI started at the imaginary intersection of the superior temporal sulcus (STS) and the lateral sulcus and traced the anterior bank of STS, extending to anterior bank of parieto-occipital sulcus (POS). The anterior borders of the medial part of the ROI were formed by the extension of the central sulcus and POS, respectively. The ventral border on the medial side traced the dorsal bank of the anterior POS, and the imaginary extension of POS until it intersected the cingulate sulcus.

To parcellate the frontal and the parietal ROIs based on their connectivity with the rest of the brain, a connectivity matrix was constructed with dimensions (number of vertices in ROI × number of vertices in the hemisphere). With this information, a symmetric cross-correlation matrix called similarity matrix was derived in which *x*(*i,j*) represented how similar the connectivity profile of the *i*th vertex was to the connectivity profile of the *j*th vertex. We then submitted the similarity matrix to a data-driven AP clustering algorithm (Frey and Dueck 2007). This algorithm parcellated the frontal and the parietal cortices, which were then back projected onto the cortical surface of the macaque brain to establish their spatial location.

While popular clustering algorithms such as k-means require the user to make an a priori declaration of the number of clusters, AP clustering independently identifies a number of clusters within a given dataset. This is important when working with data such as resting state fMRI, as its resolution might influence the number of clusters one can identify. Another advantage of AP clustering, again in contrast to *k*-means approaches, is that the algorithm identifies a single data point within each cluster—the so-called exemplar—that serves as the representative data point of the cluster. Using the connectivity profile of the exemplars, rather than the averaged or principal time course of all vertices in a cluster provides the highest signal-to-noise for further analyses. The AP algorithm, however, does require the user to specify a “preference” parameter, which when set to the same value for each data point (flat distribution across data points) ensures that there is no prior liking or affinity towards any of the data points to become exemplars. That in turn, determines how stringent the algorithm should be when deciding on separating a cluster into two. If the values of our similarity matrix *S* ranged from min(*S*) to max(*S*), then the preference value was set at min(*S*) − (max(*S*) − min(*S*)). This value results in a suitable balance between specificity in the data and an interpretable number of clusters. We have discussed three clustering validation measures, Davies–Bouldin measure, gap criterion, and silhouette measure, that demonstrate the suitability of using min(*S*) − (max(*S*) − min(*S*)) as the preference value (supplementary material, section “clustering solution validation”). Furthermore, we argue that irrespective of the underlying number of clusters, functional families used to study the principles of organization can be observed in hierarchical clustering results (supplementary material, section “preference value and stability of functional families”).

### Connectivity matrix and fingerprints

Based on the results of the frontal and parietal clustering analyses, we constructed a connectivity matrix with correlation coefficients of each of their relative exemplars. The correlation coefficient values in this matrix represented the strength of the frontal–parietal connection between exemplars, and hence between the parcels. The rows of the connectivity matrix can be considered as the parietal connectivity fingerprint (Passingham et al. 2002) of each frontal exemplar, and the columns as the frontal connectivity fingerprint of each parietal exemplar. This connectivity matrix formed the basis of all subsequent analyses.

Preprocessed data, results, and analysis code will be made available from the Donders Institute data repository (https://data.donders.ru.nl/). Links are available on the lab’s website (http://www.neuroecologylab.org).

### Hierarchical clustering and affinity matrices

The affinity propagation algorithm will return, as do many other parcellation approaches, a set of parcels, each equal in weight to the others. This final result does not describe on its own which parcel separations are dominant, and which are secondary. To elucidate a hierarchical organization of parietal–frontal connectivity and to identify the families/groups of parcel exemplars that shared a similar connectivity profile, we hierarchically clustered the connectivity matrix that describes the linkage between parietal and frontal parcels. More precisely, frontal parcels were clustered based on parietal connectivity and vice versa, relying on a dissimilarity metric as defined by “Manhattan” or “city-block” distance between (frontal) rows or (parietal) columns of the matrix using the Ward linkage method, as implemented in Matlab’s (the Mathworks) Statistics and Machine Learning and Bioinformatics toolboxes. To examine the hierarchical organization principles of the frontal and the parietal cortex, branching behavior of the resulting hierarchical tree was examined incrementally, starting from the most dominant segregation in two branches up to nine branches.

Having discussed the frontal and parietal cortex organization at different levels of hierarchy, the solution with eight frontal and seven parietal branches were selected based on anatomical priors to examine if anatomical organization can be inferred from the functional connections. To summarize the connectivity between frontal and parietal branches, we constructed an affinity matrix. An affinity matrix can be considered to represent the connectivity strength of each of the eight frontal branches to each of the seven parietal branches. Each cell represents the average correlation coefficient of all of the exemplars under that branch. The affinity matrix can also been schematically represented with arrows signifying the key connections derived from the matrix.

## Electronic supplementary material

Below is the link to the electronic supplementary material.

Supplementary material 1 (DOCX 13076 KB)
