# Supplementary material for: Mapping multiple principles of parietal–frontal cortical organization using functional connectivity
Source: Brain Struct Funct. 2018 Nov 23;224(2):681–97. doi: 10.1007/s00429-018-1791-1 (PMC6420483; doi:10.1007/s00429-018-1791-1)
Supplement: Supplementary file 1 — Supplementary material 1 (DOCX 13076 KB) [file 429_2018_1791_MOESM1_ESM.docx]

**Electronic Supplementary Material**

Brain Structure and Function

**Mapping multiple principles of parietal-frontal cortical organization using functional connectivity**

Suhas Vijayakumar^a,1^, Jerome Sallet^b^, Lennart Verhagen^b^, Davide Folloni^b^,

W. Pieter Medendorp^a^, Rogier B. Mars^a,c^

^a^ Donders Institute for Brain, Cognition and Behaviour, Radboud University Nijmegen, 6525HR Nijmegen, The Netherlands

^b^ Wellcome Centre for Integrative Neuroimaging, Department of Experimental Psychology, University of Oxford, 9 South Parks Road, Oxford OX1 3UD, United Kingdom

^c^ Wellcome Centre for Integrative Neuroimaging, Centre for Functional MRI of the Brain (FMRIB), Nuffield Department of Clinical Neurosciences, John Radcliffe Hospital, University of Oxford, Headington, Oxford OX3 9DU, Oxford, United Kingdom

^1^**Correspondence**: Suhas Vijayakumar | [s.vijayakumar@donders.ru.nl](mailto:s.vijayakumar@donders.ru.nl)

**DOI**: <https://doi.org/10.1007/s00429-018-1791-1>

Table of Contents

[Preference value for Affinity Propagation (AP) clustering 3](#_Toc523884001)

[Clustering solution validation 4](#_Toc523884002)

[Preference value and stability of functional families 5](#_Toc523884003)

[Affinity matrices 6](#_Toc523884004)

[Frontal 2 – Parietal 3 6](#_Toc523884005)

[Frontal 4 – Parietal 4 6](#_Toc523884006)

[Frontal 7 – Parietal 6 6](#_Toc523884007)

[Frontal 8 – Parietal 8 6](#_Toc523884008)

[Region of interest (ROI) definition 8](#_Toc523884009)

[Alternate parietal ROI 9](#_Toc523884010)

[Parcellation 9](#_Toc523884011)

[Principles of hierarchical organization 9](#_Toc523884012)

[Results of the left hemisphere 10](#_Toc523884013)

[Parcellation 10](#_Toc523884014)

[Principles of hierarchical organization 11](#_Toc523884015)

[Frontal cortex 11](#_Toc523884016)

[Parietal cortex 12](#_Toc523884017)

[Principles of large-scale organization 13](#_Toc523884018)

# Preference value for Affinity Propagation (AP) clustering

The AP clustering algorithm requires the user to specify a “preference” parameter, which determines how stringent the algorithm should be when deciding on separating a cluster into two. If the values of our similarity matrix *S* ranged from *min(S)* to *max(S)*, then we show the resulting clusters when the preference value was set at *min(S) – (max(S) – min(S))*, *min(S)*, and *median(S)* (Supp. Fig. 1). A cluster from a stringent preference condition tended to split into multiple clusters when using a more lenient preference condition, indicating that the parcellations are all based on meaningful signal in the data. We used three clustering validation criteria to evaluate the suitable number of clusters.


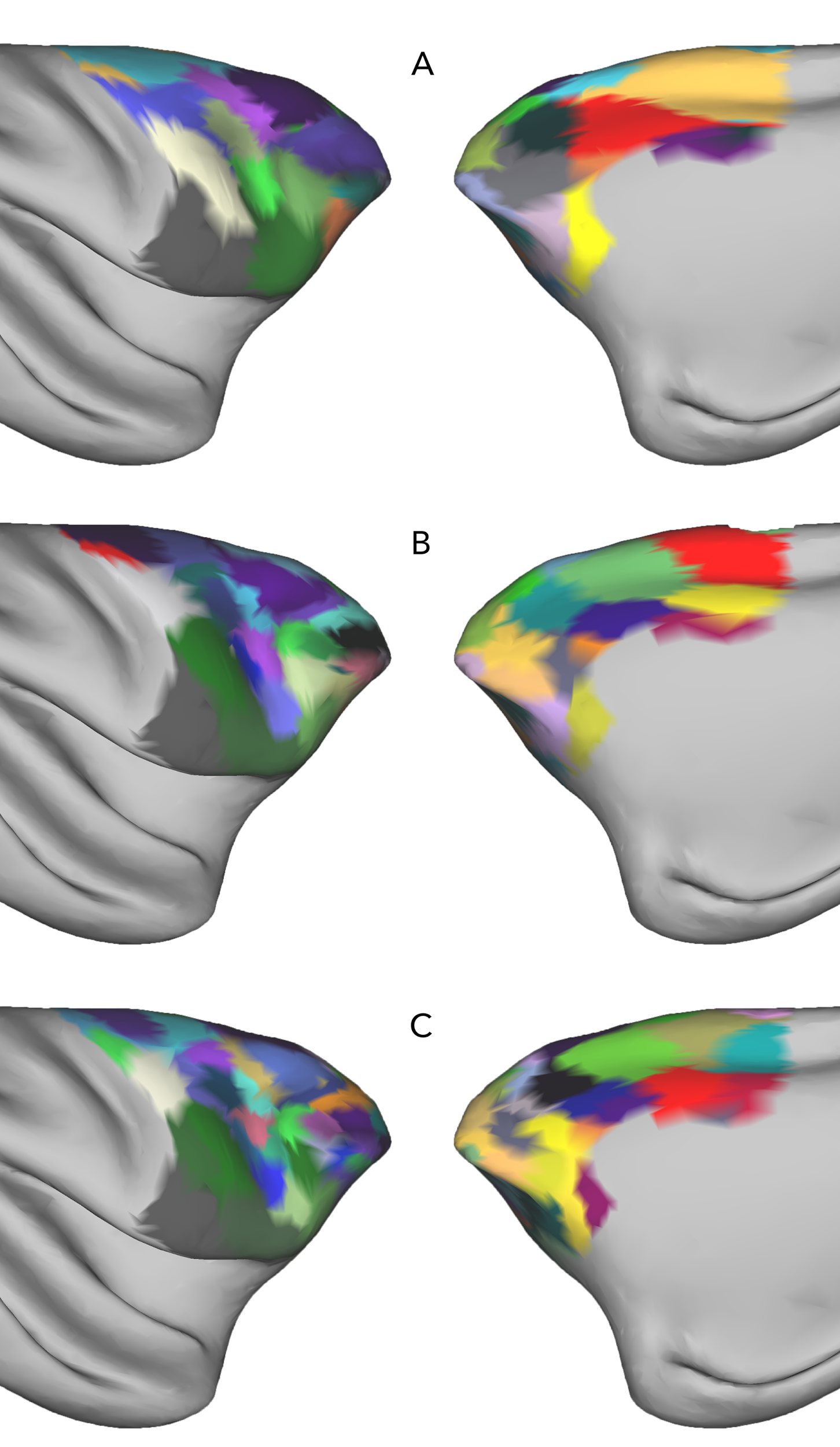


**Supplementary Fig 1** Parcellation results of the frontal cortex based on ipsilateral connectivity. Affinity propagation clustering provides different clustering results for varying preference values. If *S* is the similarity matrix, preference values were set to (**a)** *min(S) –* (*max(S) – min(S)*), resulting in 26 frontal clusters (**b)** *min(S),* resulting in 40 clusters and **(c)** *median(S)*, resulting in 67 frontal clusters

# Clustering solution validation

To identify a reasonable clustering solution and select a suitable preference parameter, we performed agglomerative clustering using ward linkage on the frontal and parietal regions of interest to result in 1 to 100 clusters. We used the Davies-Bouldin measure, gap criterion, and silhouette measure to identify suitable number of clusters (Supp. Fig. 2). They were implemented using the inbuilt Matlab function “evalclusters”. The number of clusters with minimum Davies-Bouldin value, and maximum gap criterion and silhouette values indicate the best solution. Although, the three measures seem to differ in their final solution, one can identify a range of these values where they seem to agree that the clustering solutions are reasonable. For example, in the case of right frontal clusters (Supp. Fig. 2, top panel), Davies-Bouldin value achieves local minima at 26 and 62 cluster solutions. Gap measure values remain comparable between 22 and 64 cluster solutions. Silhouette measure remains comparable between 16 and 35 cluster solutions. The solution of AP clustering, with a preference value of min(S) – (max(S) – min(S)) yielded 26 cluster solution, which lies within the range of “good” cluster solutions mentioned above.


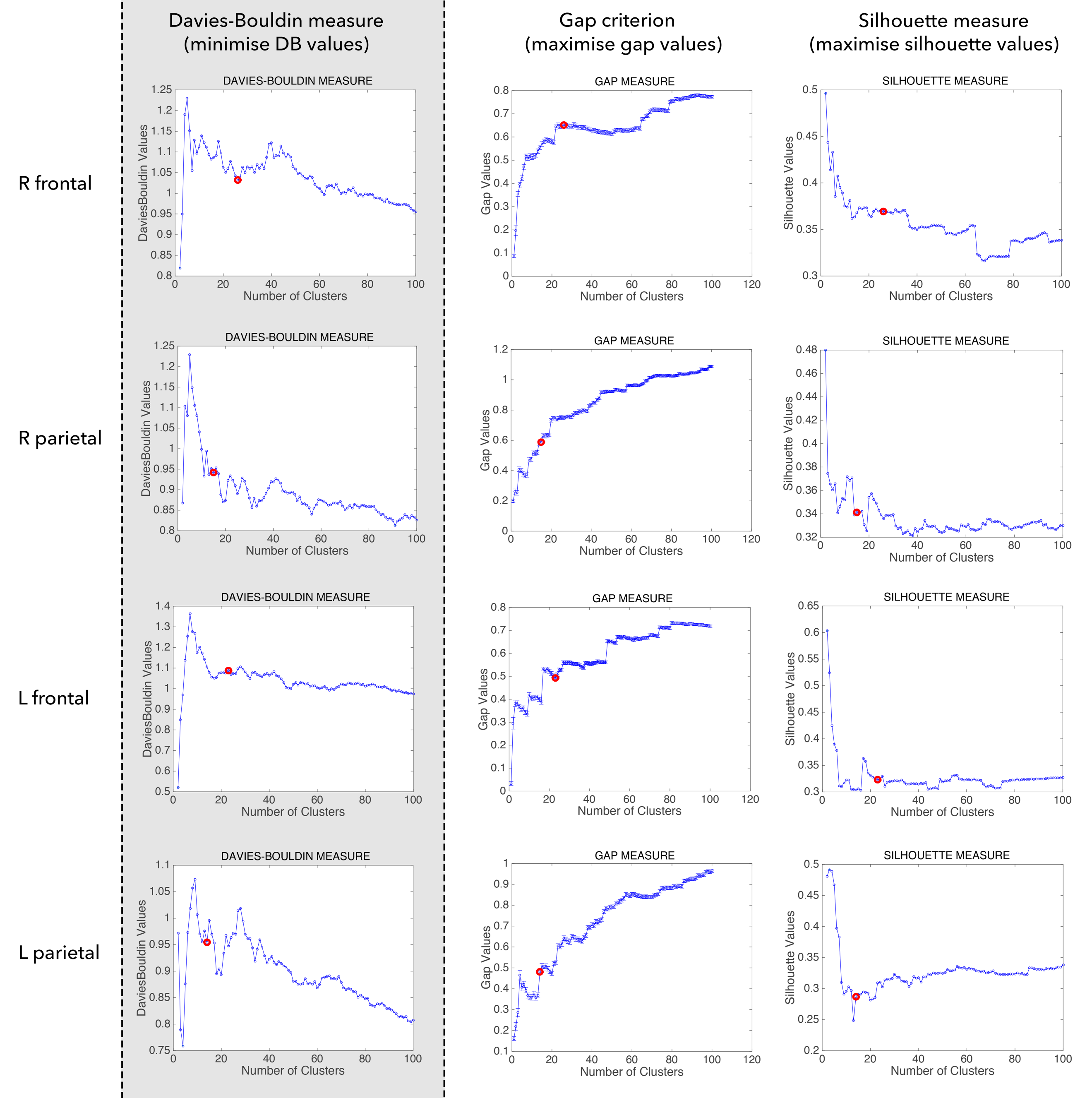


**Supplementary Fig 2** Results of clustering solution validation. Davies-Bouldin measure, gap criterion, and silhouette measures were computed for 1 to 100 clusters of agglomerative clustering performed on the frontal and parietal regions of interest. Solution obtained by using a preference value of min(S) – (max(S) – min(S)) is shown in red for each case.

# Preference value and stability of functional families

We have shown the parcellation results for three “preference” parameters in supplementary figure 1. Our aim however, was to identify the principles of parietal-frontal organization based on patterns of functional connectivity at the level of functional families, and not parcellating the two regions of interest. Parcellation step only served as a preprocessing step to reduce the complexity in the data and identify exemplars that best represented the connectivity pattern of a family of regions. We argue that irrespective of the underlying number of clusters, the functional connectivity pattern of families of regions should remain the same.

We tested the stability of these functional families, irrespective of the underlying number of clusters, in the right frontal region of interest (ROI) as proof of concept. While a preference value of *min(S) – (max(S) – min(S))* resulted in 26 frontal clusters, *min(S)* resulted in 40, and *median(S)* resulted in 67 clusters. We used the exemplars from these results and constructed connectivity fingerprints for each exemplar with the 15 exemplars of the parietal cortex (parcellated with preference = *min(S) – (max(S) – min(S))*). The frontal cortex was then hierarchically clustered to produce 8 branch solution in each case. Further, to establish the similarity between these branches, we computed dice coefficients between the 8 branch solutions resulting from each of these parcellations. First, chance-level dice coefficient was calculated by randomly assigning vertices to different branches and calculating dice coefficient between the original 8 branch solution of the *min(S) – (max(S) – min(S))* parcellation, iterated a 1000 times, and found average chance-level dice coefficient to be 0.125 (range = 0.0988 to 0.1509). Results showed that the average dice coefficients between 8 branches from *min(S) – (max(S) – min(S))* parcellation and *min(S)* parcellation was 0.6334, between *min(S) – (max(S) – min(S))* and *median(S)* was 0.5546, and between *min(S)* and *median(S)* was 0.3825, all of which, were above chance-level.

Having established the stability of functional families despite difference in underlying number of clusters, we proceeded with *min(S) – (max(S) – min(S))* results for further analyses, which strikes a suitable balance between specificity in the data and an anatomically interpretable number of clusters.


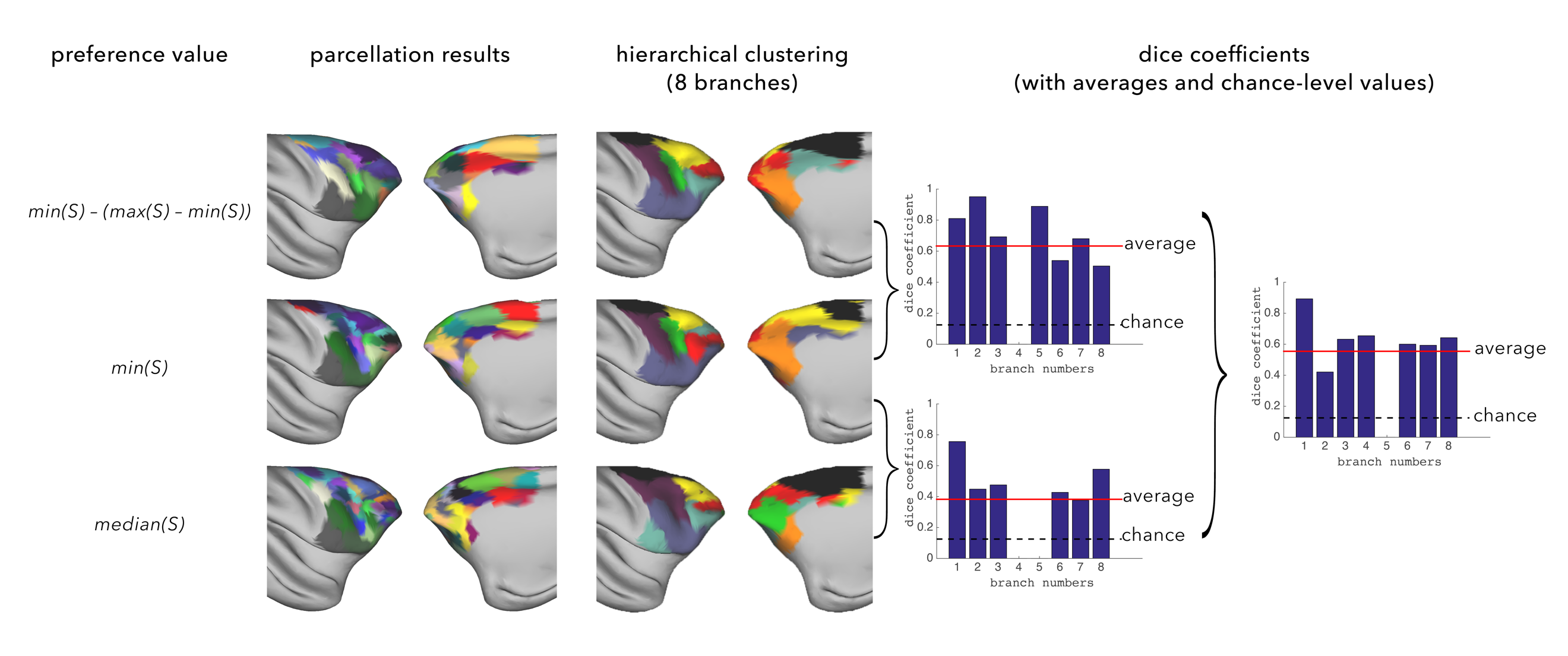


**Supplementary Fig 3** Stability of functional families. Preference parameter set to values in the first column (*S*:= similarity matrix). Parcellation results of AP clustering of the right frontal ROI for each preference value. The 8 branch solution from hierarchical clustering performed on the frontal exemplars. Dice coefficient values demonstrating similarity between functional families, despite containing different number of underlying clusters.

# Affinity matrices

Organization of the frontal and parietal cortices could be studied at multiple levels ranging from individual areas to large-scale functional networks. We aimed to understand the parietal-frontal organization at the level of functional families, which consist of multiple parietal regions with similar connectivity profiles with the frontal cortex, and vice versa. Hence, an appropriate number of branches were selected based on exploration as explained below (Supp. Fig. 4).

## Frontal 2 – Parietal 3

At the level of 2 frontal branches and 3 parietal branches, the organization was primarily driven by frontal regions that showed positive correlation with regions in the parietal cortex and by regions that mainly showed negative correlation with the regions in the parietal cortex. Due to averaging of the correlation coefficients of multiple exemplars, the strongest correlation observed was less than 0.2, whereas some of the exemplars in the connectivity fingerprints figure (Fig. 2. main text) showed correlations of over 0.4. Hence, we considered more number of branches.

## Frontal 4 – Parietal 4

At the level of 4 frontal branches and 4 parietal branches, we observed that the dorsal premotor branch showed preferential connectivity anterior parts of the parietal cortex, and the dorsal prefrontal and periarcuate branch showed stronger connectivity with the superior parietal lobe (SPL), indicating the presence of a core-shell organization. The clusters in the ventral premotor branch are spatially discontinuous and showed stronger connectivity with the posterior SPL. Frontal polar branch and the limbic regions showed weak overall connectivity with the parietal cortex. Note that even though the principles of organization begin to emerge at this stage, we are unable to completely dissociate the contribution specific functional families due to discontinuous clusters. And averaging connectivity of the exemplars in a branch results in smoothed connectivity patterns. Hence, we explored further.

## Frontal 7 – Parietal 6

We examined the 7 branch solution for the frontal cortex and the 6 branch solution of the parietal cortex. At this level, we observed that the ventral branch showed preferential high connectivity with the anterior parts of the inferior parietal lobe (IPL). The periarcuate branch showed stronger connectivity with the anterior SPL. The dorsal prefrontal branch showed stronger connectivity with the posterior SPL and IPL. And due to the averaging of the exemplars that span most of the premotor cortex, we see stronger connectivity of the region with both branches that span SPL and anterior parts of IPL. This was also the primary reason to select higher number of branches.

## Frontal 8 – Parietal 8

The first and the second parietal branch of the 8 branches solution (columns 1 and 2 of the corresponding affinity matrix in Supp. Fig. 4), showed similar connectivity pattern with that of the frontal cortex. Both had comparatively stronger connectivity with the dorsal premotor and sensorimotor areas, followed by comparatively strong connectivity with the dorsal prefrontal areas. Given that these two branches contained a single cluster, we were now studying connectivity profiles of individual clusters. So, we decided to select 7 branch solution for the parietal cortex, and 8 branch solution for the frontal cortex to construct a summary affinity matrix that highlighted the principles of brain organization (Fig. 6, main text).

An increase in the number of branches provides little further insight in to the organization of the parietal-frontal network at the level of connectional families. As 8 branch solution of the parietal cortex includes two branches with lone clusters, we utilized 8 frontal and 7 parietal branch solutions to make the summary figure highlighting the results (Fig. 6, main text).


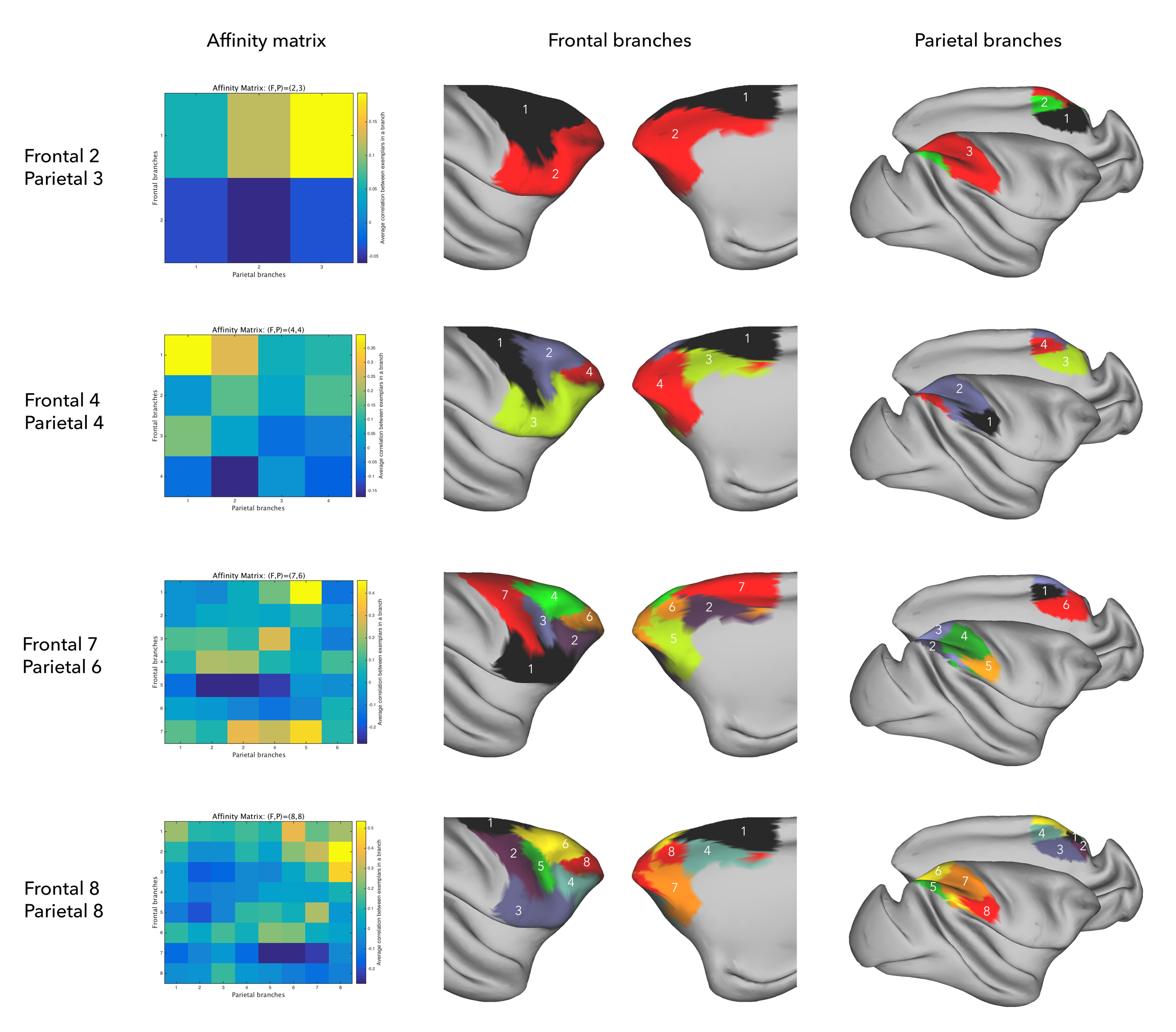


**Supplementary Fig 4** Affinity matrices at different levels of frontal and parietal branches are shown along with the projection of these branches on an inflated macaque brain. Numbers on the projection indicate branch numbers, and the respective row/column numbers in their corresponding affinity matrix.

# Region of interest (ROI) definition

A detailed description of the frontal and parietal ROI definition is given in the main text. Refer to Supplementary Figure 5 for the relevant sulcal anatomy and ROIs used for the analyses. We also demonstrate the robustness of results for an alternative ROI definition that includes areas of the somatosensory cortex in the anterior part of the superior parietal lobule (SPL) (Suppl. Fig. 5C) in the following section.


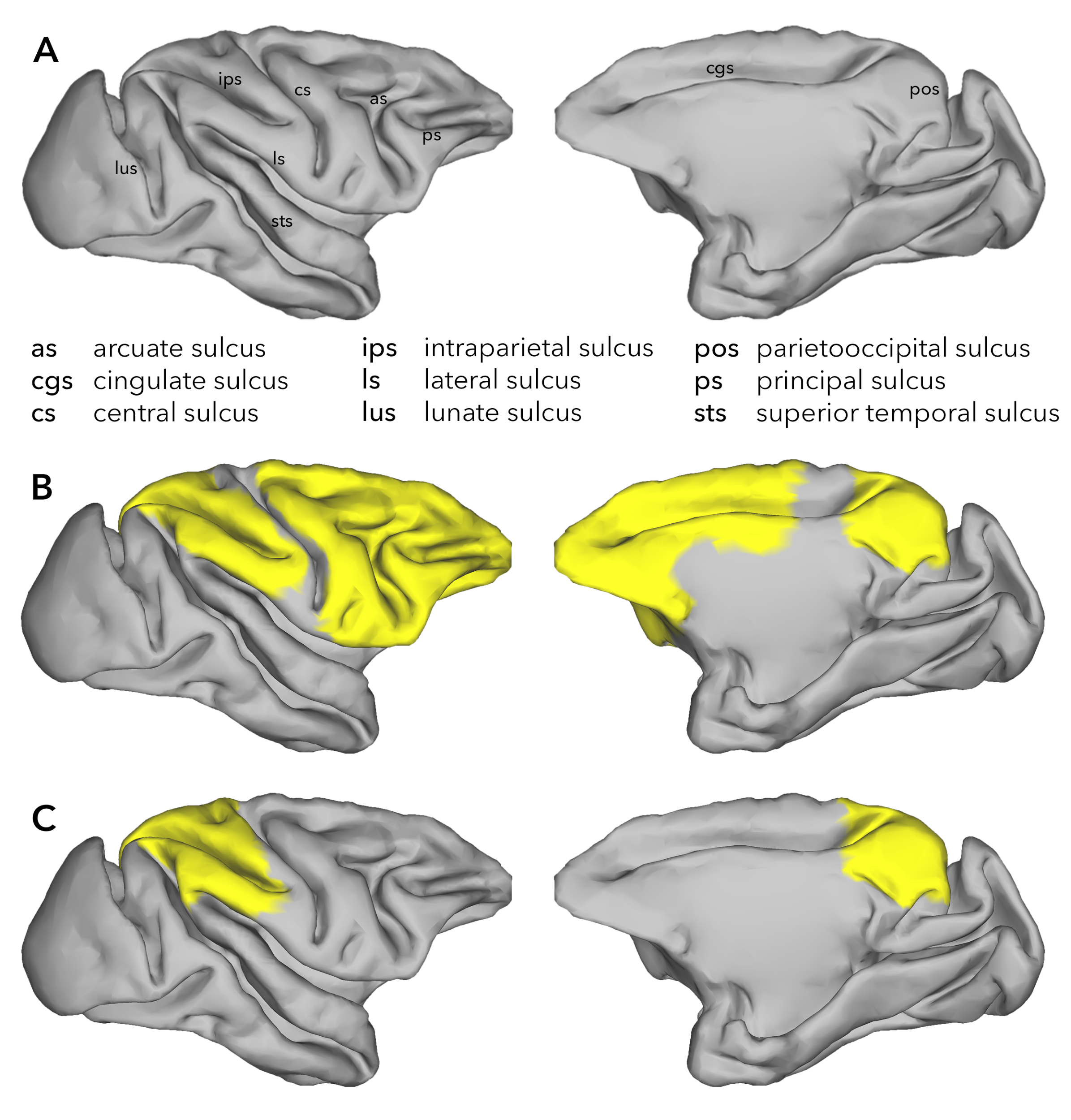


**Supplementary Fig 5** **(a)** Some of the main sulcal landmarks in the macaque brain are labelled. **(b)** Parietal and frontal ROI masks used for parcellation. **(c)** Alternative parietal mask used to show the consistency of overarching results

# Alternate parietal ROI

## Parcellation

Parietal clusters were identified using AP clustering algorithm for the alternative ROI (Supp. Fig. 5C), based on its connectivity with the ipsilateral hemisphere. Results (Supp. Fig. 6) included clusters numbered P1, P2, P3, P4, P5, and P7 along the medial side of the SPL (V6A, PEci, PGm). Clusters P6, P9, and P12 were in the territory of dorsal SPL (PEc); clusters P16, P11, and P14 were part of the ventral SPL (PE); cluster P18 overlapped with anterior inferior parietal lobule (IPL) (PF) and cluster P17 overlapped with the middle IPL (PFG). Clusters P15, P8, P10, and P13 corresponded to the posterior IPL (PG, Opt).

These results differed from the parcellation results in the main text in two ways. First, the territory that was included in the new ROI was assigned to two new clusters: cluster P12 at the rostral-dorsal end of the SPL and cluster P17 at the border of the inferior parietal and superior temporal cortex. Second, cluster P13 and part of cluster P10 from the new parcellation were combined into a single cluster, cluster P10 in the original parcellation.

## Principles of hierarchical organization

Hierarchical clustering of the parietal exemplars based on their connectivity with the frontal exemplars in the main (Fig. 1, main text) and alternative (Supp. Fig. 6) parcellations showed remarkably similar organization, resulting in families similar to the ones discussed in the main text. The alternative parcellation’s superior parietal cluster P12 groups with the posterior IPL and medial parietal, which also tended to cluster together in the main results. IPL/superior temporal cluster P17 groups together with Opt. The main groupings of the hierarchical trees are similar, although the different families tend to be ordered differently.


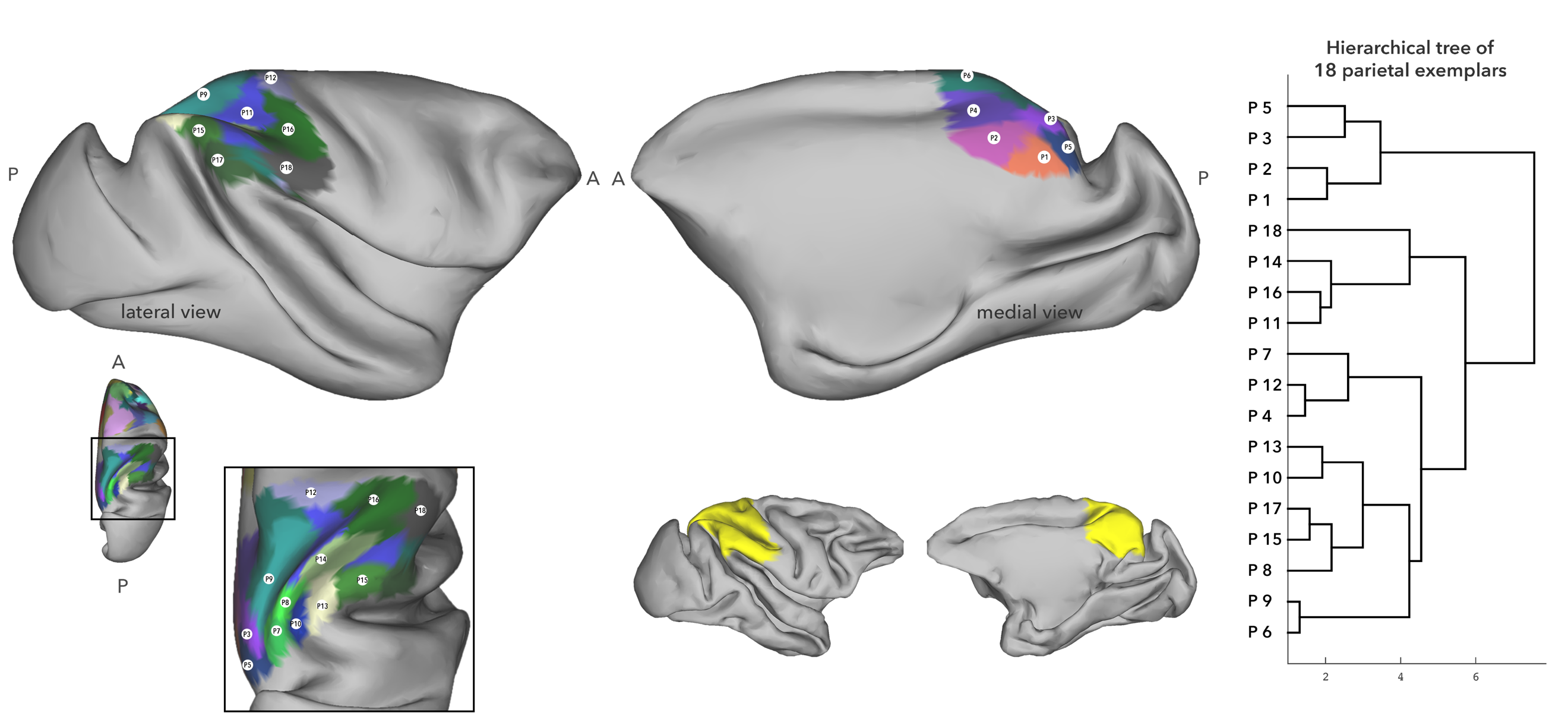


**Supplementary Fig 6** Results of AP clustering of the alternative parietal ROI based on its connectivity strength within the hemisphere, back-projected on an inflated macaque brain. The cluster numbers mark the position of the exemplar as identified by the AP clustering algorithm. Hierarchical clustering of the parietal exemplars based on their connectivity with the frontal exemplars is given on the right-hand side

# Results of the left hemisphere

## Parcellation

A detailed description of the definition of the frontal and the parietal ROIs for the right hemisphere is discussed in the main text. ROIs for the left hemisphere followed the same sulcal boundaries as the right hemisphere, drawn using Workbench. Parcellation using AP clustering with a preference parameter of *min(S) –* (*max(S) – min(S)*) resulted in 23 clusters for the left-frontal ROI and in 14 clusters for the left-parietal ROI (Supp. Fig. 7). Connectivity fingerprints of the exemplars are presented in supplementary figure 8. Similar to the right hemisphere, here we label the resulting clusters based on their overlap with previously published atlases only to provide a broad understanding of the parcellation results, and make the clusters more tractable.

On the lateral surface of the frontal cortex, clusters 1 and 2 overlapped with the ventral premotor cortex and the macaque homolog area 44. The periarcuate clusters 7, 11, and 10 were in the territory of area 8. Clusters 9 and 4 overlapped with dorsal premotor cortex, with cluster 9 also spanning the pre-SMA region on the medial side. Clusters 13 was part of the dorsal PFC (area 9) and clusters 6 and 3 were in the territory of ventral later PFC, cluster 21 formed a discontinuous cluster and spanned both these regions. But the exemplar was situated in the territory of medial dorsal PFC (area 9). Clusters 16 and 12 overlapped with dorso-lateral PFC (area 46). Cluster 15 corresponded to the fronto-polar cortex. And clusters 14, 8, 5,20, and 17 were in the territory of the orbitofrontal cortex (areas 11, 13, 47/12). One the medial side, clusters 19 and 23 were in the territory of the ACC (area 14 and area 32 respectively). And cluster 22 corresponded to SMA. Note that cluster 9 also spans the pre-SMA area, but the exemplar was in the dorsal premotor region (F2).

On the lateral side of the parietal cortex, cluster 1 spanned parts of both SPL and IPL, but its exemplar was in the territory of anterior IPL. On the SPL, cluster 2 occupied area PEa, on the anterior bank of the inferior parietal sulcus (IPS). Posterior to that, clusters 4 and 7 largely overlapped with regions VIP and MIP respectively. Cluster 8 was situated dorsal to that, in the territory of area PEc. Cluster 3 on the inferior bank of IPS was in the territory of LIP and PG. Further posterior to that, cluster 6 was in the territory of Opt. On the SPL, cluster 5 overlapped with region PE. On the medial side, cluster 10 was situated in the territory of PE. Ventral to that, clusters 12 and 14 overlapped with PGm, with cluster 12 also spanning parts of PEc. Posterior to these clusters, clusters 11, 13, and 9 overlapped with region V6a.


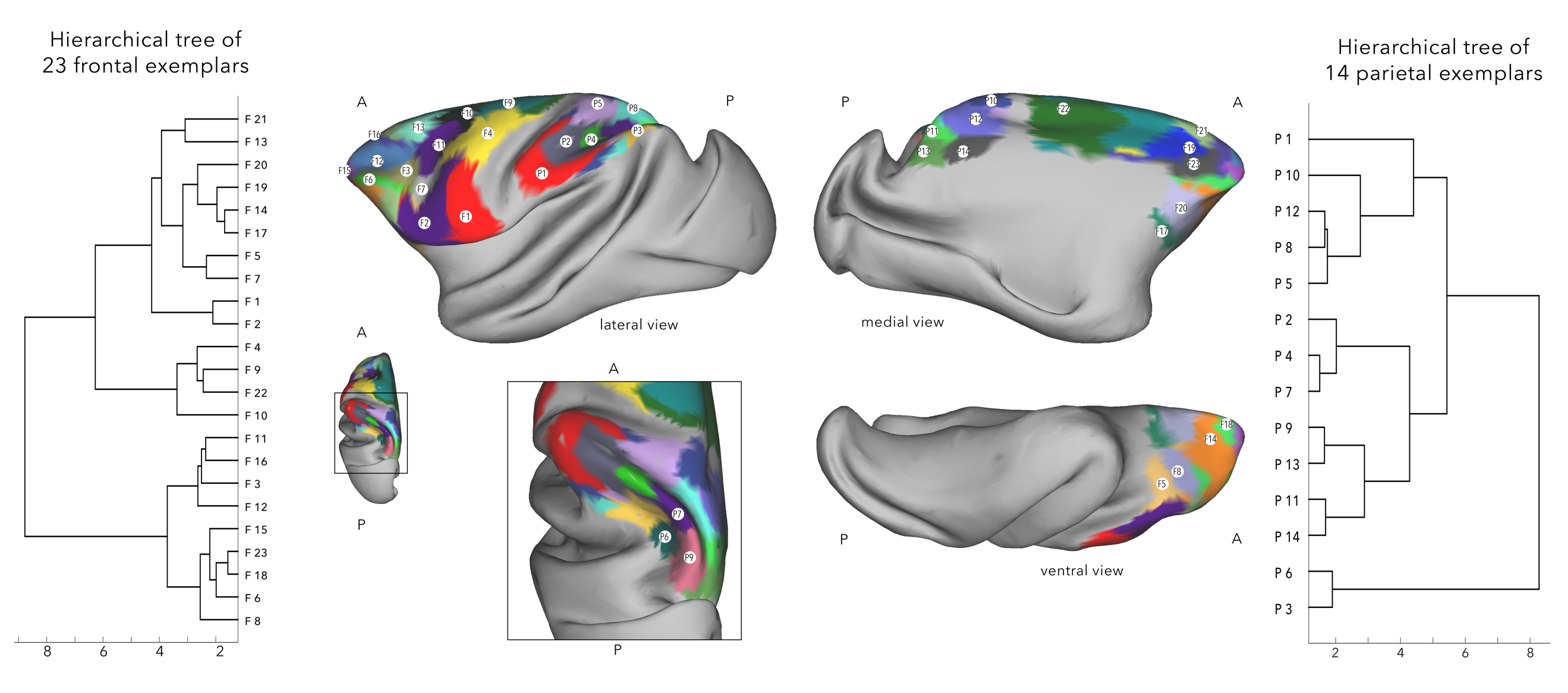


**Supplementary Fig 7** Results of AP clustering of the parietal and the frontal ROIs of the left-hemisphere based on their respective connectivity strength within the hemisphere, back-projected on an inflated macaque brain. The cluster numbers mark the position of the exemplar as identified by the AP clustering algorithm. Hierarchical clustering of the frontal exemplars based on their connectivity with the parietal cortex is given on left and that of the parietal exemplars based on their connectivity with the frontal cortex is on the right side of the parcellation results.


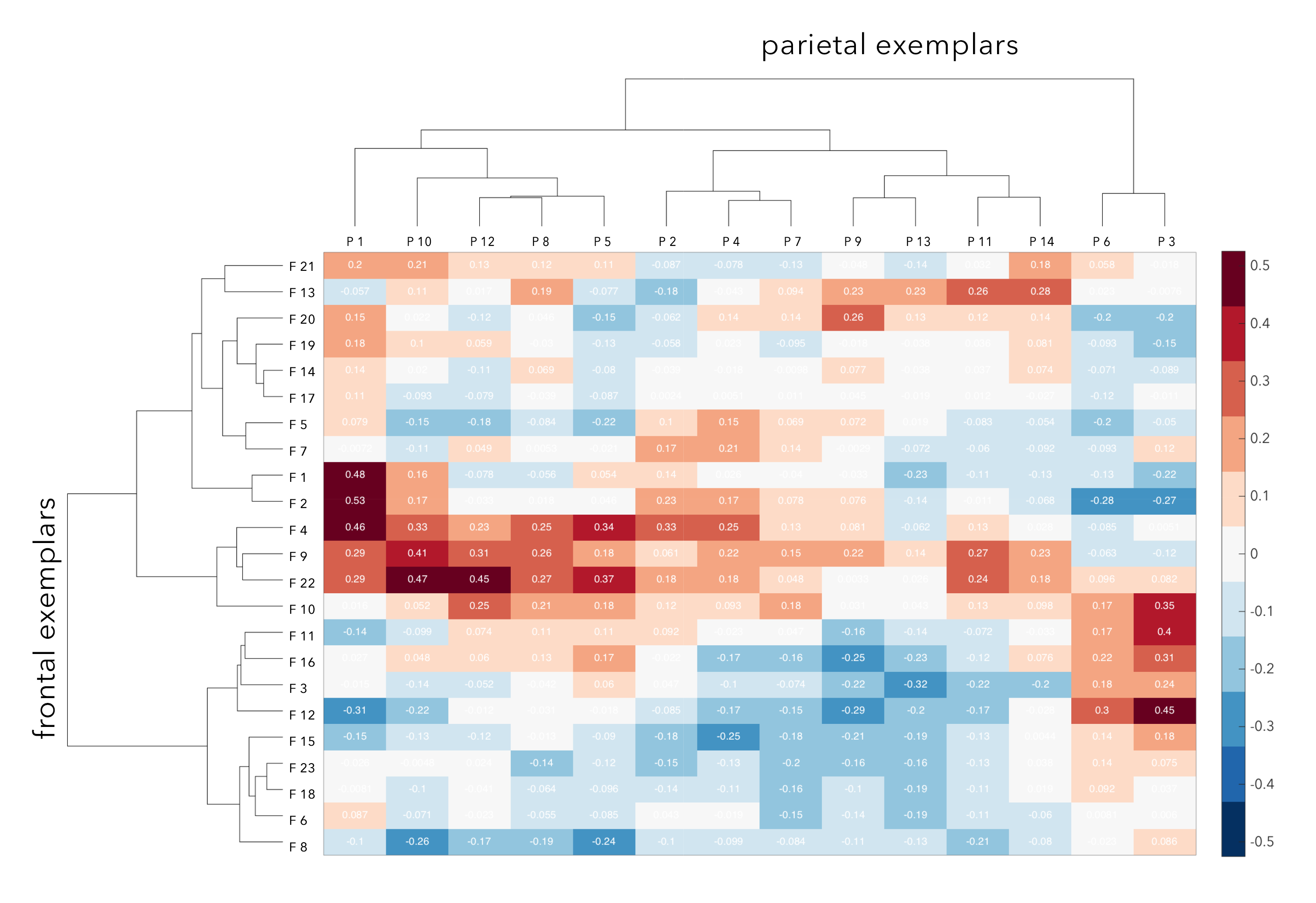


**Supplementary Fig 8** Connectivity fingerprints of the frontal (rows) and the parietal exemplars (columns) of the left hemisphere, along with their hierarchical clustering outline. Values indicated in each cell correspond to the correlation value of the corresponding frontal and parietal exemplars, as shown in supplementary figure 5. Colorbar ranges from the lowest to the highest correlation value of the exemplars

## Principles of hierarchical organization

### Frontal cortex

Supplementary figure 9 contains the results of hierarchical clustering on the left frontal exemplars based on their connectivity the left parietal exemplars. The branching of the frontal cortex at the level of two branches was driven by regions with widespread strong connectivity with the parietal cortex, and by regions with overall weak parietal connectivity, but comparatively stronger focal connectivity. The regions with widespread, strong connectivity were further divided into ventral premotor and ventro-lateral prefrontal regions with strong lateral parietal connectivity, and into a branch with dorsal premotor cortex and the SMA, which showed strong medial parietal connectivity (see step 2 to 3 branches). The regions with overall weak, but strong focal connectivity with the parietal cortex were divided into dorsal bank of the principal sulcus – dorsal prefrontal and periarcuate regions, and central bank of the principal sulcus and the fronto-polar region (5 – 6 branches) based on the preferential connectivity of the later regions with the posterior IPL and Opt.

The lateral branch of the 3 branch solution was further divided in to ventral premotor branch that showed stronger connectivity with the anterior parts of the parietal cortex and Opt, and a branch with discontinuous clusters that showed moderate connectivity with the rest of the parietal cortex (3 to 4 branches). The dorsal lateral prefrontal cortex separated from the branch that showed moderate connectivity with the parietal cortex based on their preferential connectivity the medial regions (4 to 5 branches). The lateral branch of 3 branch solution separated into dorsal premotor and SMA, and single periarcuate cluster (F10) based on its preferential connectivity with the posterior parietal regions (6 – 7 branches). The discontinuous clusters that formed a branch based on overall moderate connectivity, further divided into regions with slightly stronger connectivity with the IPS, and regions with overall weak connectivity (7 - 8 branches). At the level of 9 branches, we have branches splitting into individual regions. Hence to retain focus on the pattern of connectivity displayed by functional families, 8 branch solution was used to construct the affinity matrix.


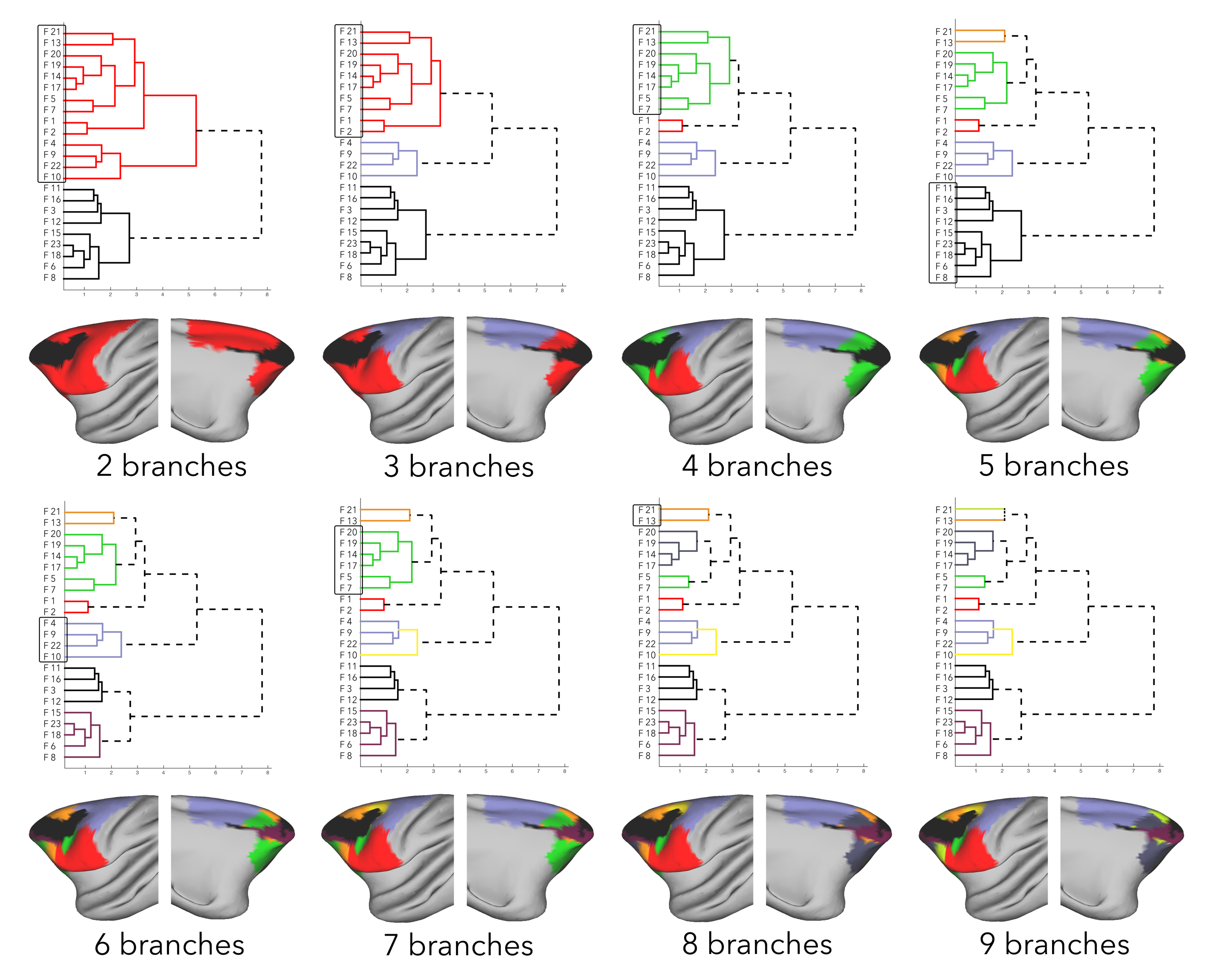


**Supplementary Fig 9** Hierarchical clustering results of the left-frontal exemplars based on their left-parietal connectivity strength. Exemplars (by extension, parcels) that belong to a branch are overlaid on the surface of the cortex in the same color, and the branch that splits from its cluster in the following step is marked with a box

### Parietal cortex

Supplementary figure 10 contains the hierarchical clustering results of the parietal exemplars based on their connectivity with the left frontal exemplars. The two branch solution was driven by regions with preferential connectivity with the frontal-polar and dorsolateral frontal cortex, and by regions with more widespread connectivity with the frontal cortex. The branch with widespread connectivity was divided into regions with strong connectivity with the medial prefrontal cortex, and into regions with strong connectivity with the lateral frontal cortex (2 - 3 branches). The branch with lateral connectivity was then divided into a branch with one cluster (P1) based on anterior most cluster’s preferential connectivity with the ventrolateral frontal cortex, and into a branch that showed strong connectivity with the dorsolateral frontal cortex (3 -4 branches). The dorsal branch was further divided into a branch with only cluster P10 based on the strong medial connectivity with the SMA, and into a branch with regions that showed strong connectivity with the dorsal premotor cortex (6 – 7 branches).

The branch that showed relatively strong connectivity with the ventrolateral frontal cortex, was divided into a branch with regions that show strong connectivity with the ventral premotor, and into a medial parietal branch of regions in the intraparietal sulcus (IPS) that show preferential connectivity with the dorsolateral prefrontal cortex and some parts of orbitofrontal cortex (4 – 5 branches). The medial parietal branch was then divided based on a regions with preferential connectivity with the dorsolateral prefrontal cortex, and into regions with preferential connectivity with the regions of the orbitofrontal cortex that extend to the medial side (5 – 6 branches). The branch with IPS regions was divided into an anterior and a posterior branch, based on the preferential connectivity of the anterior branch with the posterior regions of the frontal cortex, and on the connectivity of posterior IPS regions with the relatively anterior frontal regions (7 – 8 branches). The branch from the two branch solution that showed selective connectivity with the frontopolar and dorsolateral prefrontal cortex show similar connectivity pattern with the frontal cortex. But they were separated in the 9 branch solution based on the cluster in territory of LIP and PG showing strong connectivity with the area 8 in the frontal cortex (8 – 9 branches).


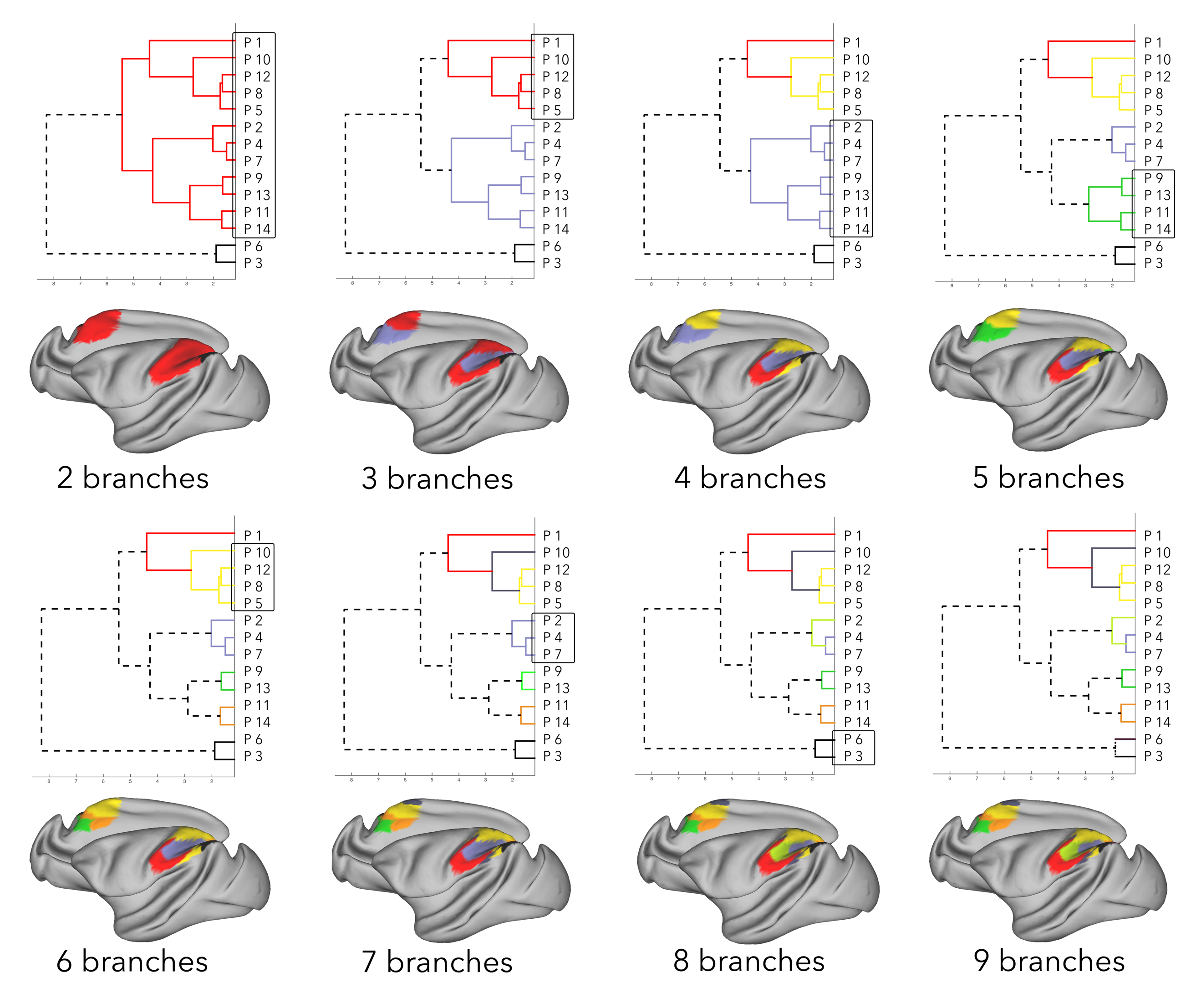


**Supplementary Fig 10** Hierarchical clustering results of the left-parietal exemplars based on their left-frontal connectivity strength. Exemplars (by extension, parcels) that belong to a branch are overlaid on the surface of the cortex in the same color, and the branch that splits from its cluster in the following step is marked with a box

## Principles of large-scale organization

Just as in the right-hemisphere, hierarchical clustering results provided insight into the organization of the macaque left frontal cortex and the left parietal cortex. We observed both dorsal-ventral organization of the premotor cortex, as well as the core-shell principle of organization in frontal-parietal networks (Supp. Fig. 11). We then created an affinity matrix that described the connectivity strength of the frontal-parietal connectional families (Supp. Fig. 12). Again, similar to the right hemisphere, affinity matrix captured prominent frontal-parietal pathways (see main text for a detailed description).


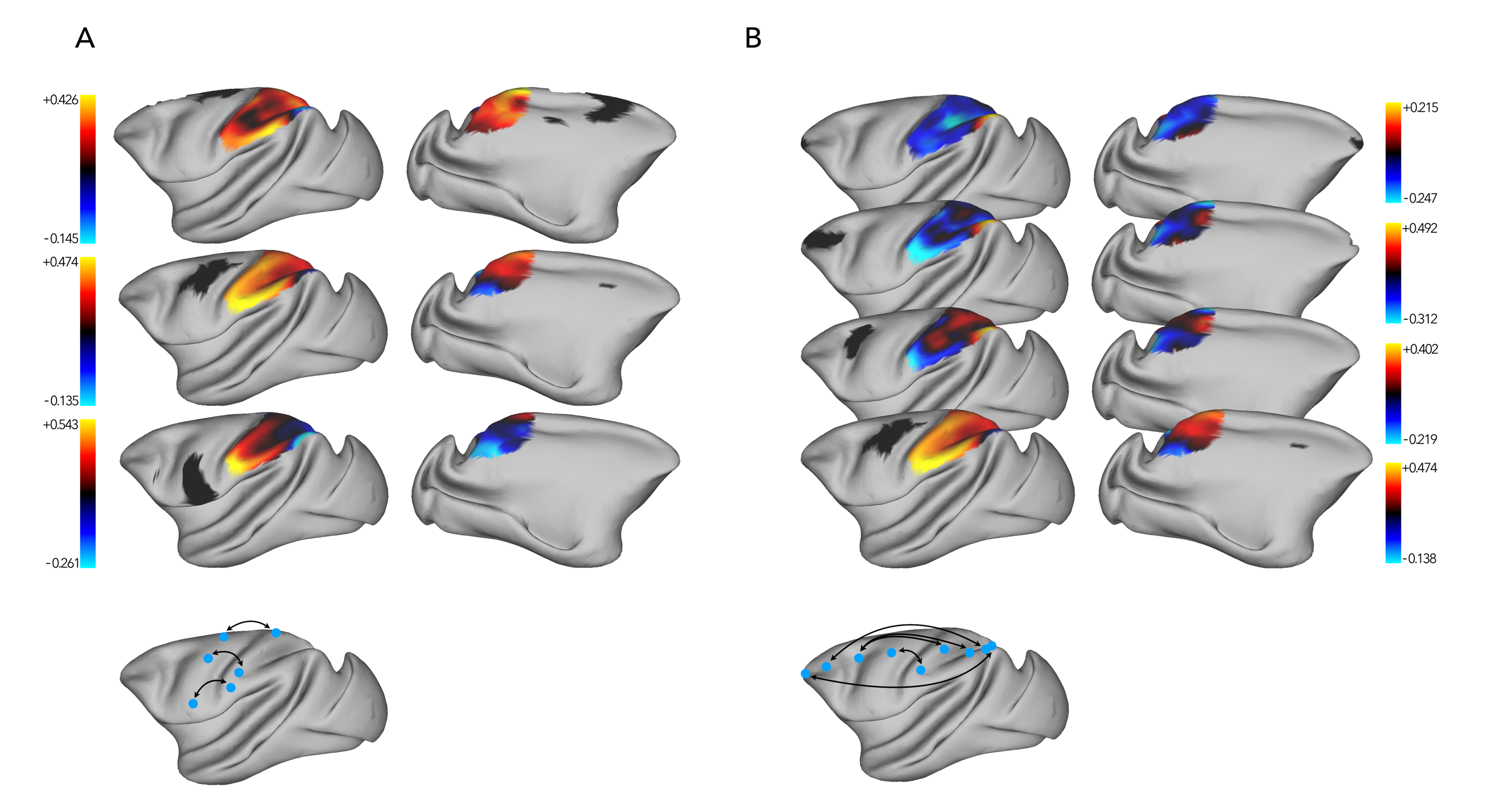


**Supplementary Fig 11** Regions in the left frontal cortex and their corresponding exemplar’s connectivity with the left parietal cortex. Black regions indicate seed areas in the frontal cortex, with the blue-red-yellow colors indicating increasing connectivity with each parietal vertex. Bottom panel shows a schematic representation of the organization of these regions **(a)** Dorsal-ventral organization of parietal connectivity with the premotor cortex. **(b)** Rostral-caudal or core-shell organization of parietal connectivity with the lateral frontal cortex. Color bars represent the connectivity correlation values of the exemplars of these seed regions. Note that the color of the ROI does not represent its within-region connectivity, but just a representation of the region


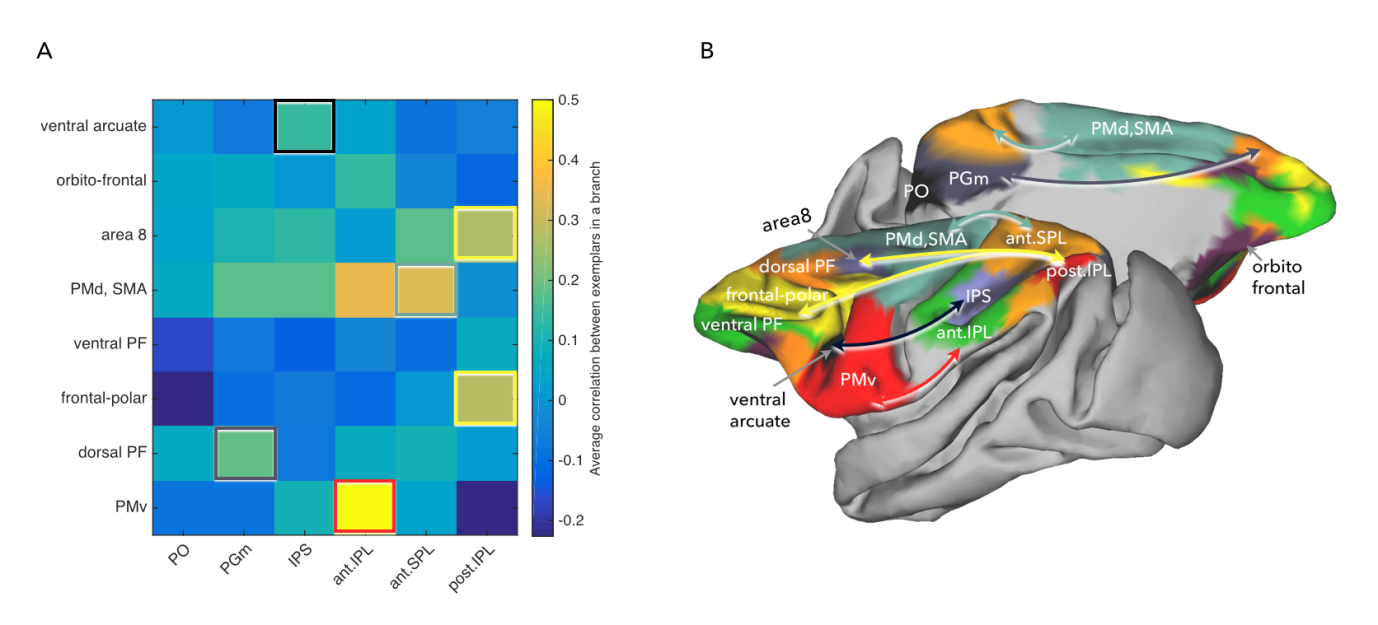


**Supplementary Fig 12 (a)** Affinity matrix of the 8 frontal hierarchical branches (rows) with that of the 6 parietal branches (columns) of the left hemisphere, indicating the average connectivity strength of the exemplars within each branch. **(b)** The branches are overlaid on a macaque brain along with arrows representing the peak connectivity between branches.

## Labelling parcellated clusters based on atlases

Labelling right frontal and parietal parcellation clusters based on the atlases of Lewis & Van Essen (2000), Paxinos, Huang, & Toga (2000), and Markov et al., (2011).

| Cluster number | Lewis & van Essen (2000) | Paxinos et al., (2000) | Markov et al., (2011) |
| --- | --- | --- | --- |
| Frontal clusters | | | |
| 1 | 24ab | 24b | 24b |
| 2 | Unassigned | Medial wall | Medial wall |
| 3 | 32 | 24a | 32 |
| 4 | Unassigned | Medial wall | 25 |
| 5 | 6DC | 6M | F3 |
| 6 | 10 | 10M | 10 |
| 7 | 9 | 9M | 9 |
| 8 | Unassigned (14r) | 25 | 25 |
| 9 | Unassigned | 10v | 10 |
| 10 | Unassigned | 13 | Unassigned |
| 11 | Unassigned (24d) | 24c | 24c |
| 12 | 6Ds | 6DR(F7) | F7 |
| 13 | 46v | 46D | 46d |
| 14 | 4 | 4 (F1) | F1 |
| 15 | 4C | 6VC (F4) | F4 |
| 16 | 13m | 13L | 12 |
| 17 | 6Ds | 6DC (F2) | F2 |
| 18 | 46p | 9/46D | 8 |
| 19 | Unassigned | 47 (12) | 12 |
| 20 | Unassigned | 44 | 8 |
| 21 | Unassigned | 47(12)O | 12 |
| 22 | 4C | 6DC (F2) | F2 |
| 23 | 4C | 6VC (F4) | F4 |
| 24 | 45 | 8AV | Unassigned |
| 25 | 12 | 44 | Unassigned |
| 26 | 6Vb | 6VC (F4) | F5 |
| Parietal clusters | | | |
| 1 | PO | PGM | PO |
| 2 | Unassigned | PGM | 31 |
| 3 | 31 | 31 | 31 |
| 4 | PO | PEC | PO |
| 5 | PO | PO | PO |
| 6 | 5D | PECg | 5 |
| 7 | VIPm | DIP | VIP |
| 8 | PIP | PEa | VIP |
| 9 | Unassigned | PEC | 5 |
| 10 | 7a | OPT | 7a |
| 11 | 5V | PEa | 5 |
| 12 | VIPI | POal | LIP |
| 13 | AIP | PEa | 5 |
| 14 | 7a | PG | 7A |
| 15 | 7t | 2 | Unassigned |

**Supplementary table 1** Exemplars of the right frontal cortex and the right parietal cortex were identified and labelled according to atlases Lewis & Van Essen (2000), Paxinos et al., (2000), and Markov et al., (2011).

# References

Lewis, J. W., & Van Essen, D. C. (2000). Mapping of architectonic subdivisions in the macaque monkey, with emphasis on parieto-occipital cortex. *The Journal of Comparative Neurology*, *428*(1), 79–111. https://doi.org/10.1002/1096-9861(20001204)428:1<79::AID-CNE7>3.0.CO;2-Q

Markov, N. T., Misery, P., Falchier, A., Lamy, C., Vezoli, J., Quilodran, R., Gariel, M. A., Giroud, P., Ercsey-Ravasz, M., Pilaz, L. J., Huissoud, C., Barone, P., Dehay, C., Toroczkai, Z., Van Essen, D. C., Kennedy, H., & Knoblauch, K. (2011). Weight Consistency Specifies Regularities of Macaque Cortical Networks. *Cerebral Cortex*, *21*(6), 1254–1272. https://doi.org/10.1093/cercor/bhq201

Paxinos, G., Huang, X. F., & Toga, A. W. (2000). The Rhesus Monkey Brain in Stereotaxic Coordinates.
